# Spirit of the Foreign Periodicals

**Published:** 1835-01-01

**Authors:** 


### Spirit of the Foreign Periodicals, &c.

On the Employment of large Doses of the Tartrate of Antimony, after severe Wounds and other Injuries.

M. Franc, in his prize memoir, has brought together a multitude of cases of severe injury, to demonstrate the salutary effects of this very powerful remedy. The cases have been drawn chiefly from the cliniques of MM. Delpech and Lallemand, in the Montpellier hospitals, and are valuable alike for the accuracy of their histories and the results of their treatment. The medicine was usually given in full doses—8 or 12 grains being dissolved in water, and a fourth part being given every half-hour, till the muscular energy and the arterial and nervous excitement were subdued; and then smaller doses were continued, at greater intervals, to keep up the contra-stimulant effects. Several cases of simple and compound dislocations, in which the reduction was effected with unusual facility, after the exhibition of the tartrate, are mentioned; and, indeed, it was in this class of accidents, and in injuries of the head, that the good effects of the antimonial treatment are chiefly insisted upon. In almost every example, the inflammatory and nervous symptoms were prevented by the timely employment of the tartrate, either with or without the previous abstraction of blood; and even when a train of distressing symptoms had set in before the patient applied for assistance, the relief was uniform and most striking, as soon as the "tolerance" was once fairly established. It is to be observed, however, that, on the whole, this treatment is much more effectual as a preventive than as a curative means.

M. Franc has done a service to his professional brethren, by collecting and arranging the materials of his memoir, for, although the treatment is neither novel nor unknown, we believe that it is not quite so extensively adopted as it ought to be. Many a pound of blood might be saved, if the antimonial antiphlogistic was duly appreciated on all occasions.—*D'Emploi du Tartre, &c. Montpellier*, 1834.

---

Wretched State of Midwifery in Germany.

The heading to this article we have given designedly, and it will probably be justified by our readers when they have perused the following details. The reporter of the case is the celebrated Professor Naegele, of Heidelberg.

Mad. K. aged 30, was delivered of a well-formed boy on the 5th Sept. The placenta was not, as usual, expelled, and the midwife in attendance contented herself with attaching the umbilical cord to one of the patient's thighs!! On the following day a consultation was held (we suppose M. Naegele was present), and it was decided that an attempt should be made to extract the after-birth; but, as it was found to adhere very strongly to the uterus, the attempts were discontinued, and injections of sage and camomile-flowers ordered! Next day, another ineffectual attempt was made, and in the evening of this day, symptoms of peritonitic distress began to develop themselves. On the 8th, being the third day after delivery, the following is the distressing report: "patient much depressed—skin bedewed with an offensive sweat—tongue covered with a thick, brownish crust—pulse rapid and feeble—thighs, hips, and body exhibiting a vesicular eruption, which is attended with sharp pain in the parts—the lochia scanty and extremely fetid. A third attempt was made at extraction, but in vain." Well may the Professor say that the state of his patient was now alarming; however, as good fortune would have it, this woman, after a fortnight's extreme danger, gradually recovered *in spite* of her doctors, who gravely inform us that, "at the end of three months, her health was completely restored, although, during the whole of the time, no traces of the debris of the placenta had been observed in the vaginal discharge." The preceding most worthy history is closed with some sage deductions; for example, we are advised, in cases of abortion in which the placenta is not expelled, not to attempt to extract it with "la pince à faux germe," or any such like instrument, although we have the high authority of Osiander for doing so; and that, after an accouchement, we should not continue groping with our fingers, within the uterus, for every fraction of a fragment of the placenta which we, in our ignorance, may suppose to be left behind!!

It is quite unnecessary to condemn such practice to the British practitioner—the veriest youth, who goes trembling to his "first midwifery case," might give some of his sage seniors, in other countries, a good practical lesson. The preceding case is derived from a late No. of the *Annales Cliniques de Heidelberg.*

---

### The Virtues of Sarsaparilla appreciated in Germany.

It is well known that very different estimates are formed of the medicinal value of this drug by different practitioners in this country, and that, while most surgeons laud it most highly, our physicians seem scarcely to recognize it as a remedy at all. Probably this discrepancy of opinion is attributable to the circumstance of syphilis and syphilitic affections having hitherto been deemed surgical, and not medical diseases! although they may very fairly claim the patronage of the purest Fellow of the College of Physicians, as being truly internal and constitutional in many of their varying forms and phases. The preparation of sarsaparilla chiefly used in Germany, is what is generally known there under the name of decoction of Zittman. There is a strong and a weak decoction—the former is prepared thus:

Take of sarsaparilla-root a pound—pour on it 24 pounds of boiling water, and let the infusion stand for 24 hours; a bag, containing an ounce of sugar, and the same quantity of alum, with half an ounce of calomel, and a drachm of sulphuret of mercury, is to be put into the infusion, and this is to be then slowly boiled down to two-thirds of the original quantity: to the decoction thus obtained, half an ounce of anise and fennel seeds, three ounces of senna leaves, and an ounce and a half of liquorice-root are to be added.

The weak decoction is prepared by boiling six ounces of sarsaparilla in 24 pounds of water, and then adding lemon-peel, canella bark, smaller cardamom seeds, and liquorice-root, of each three ounces. A fortnight's use of the remedy is, we are told, usually sufficient; but some cases require it to be continued for a much longer period of time. The "diete" ought to be very rigid, and Dr. Chelius, one of the warmest advocates of the medicine, advises the patients to keep their beds constantly while taking it. The physiological effects are, generally, an increase in the perspiratory, alvine, and urinary secretions. Salivation is very seldom induced, however long the decoction be taken.—*Annales de Hecker.*

---

### Extracts from the German Journals, with some Remarks on the Character of Continental Medical Literature.

1. *Pemphigus often associated with Intermittent Fevers.*

Professor Berndt has related, in Hufeland's Journal for last Nov. several cases of that rare eruptive disease, pemphigus. All the patients had been, and continued to be, subject to attacks of tertian, or other intermittent fever; and, indeed, the cutaneous affection seemed obviously to be dependent upon the same morbid state of the system as that which gave rise to the ague; for it was recurrent, although irregularly so, and it yielded to the remedies which are known to be most effectual against these, viz. cinchona and arsenic. The bullæ varied much in size in different patients; sometimes they were not larger than a pea, while, at other times, they exceeded the size of a kidney-bean.

### 2. *Sulphate of Copper as an Emetic in Croup.*

In the same German Journal, we find a communication in which this cupreous salt is strongly recommended, in the tracheal and bronchitic affections of children.

In the dose of from three to five grains, it acts very speedily as a powerful emetic, and thus promotes the expulsion of any false membrane which may be lining the air-passages. The advantage which it possesses over ipecacuan and tartar-emetic is its more prompt and certain operation, without inducing any subsequent exhaustion or profuse evacuation; accidents which are sometimes fatal in very young patients. The employment of the sulphate as an emetic, in the cases referred to, is not, as a matter of course, to be employed to the exclusion of bleeding, calomel, and other means usually adopted in inflammatory diseases.

Before dismissing our German contemporary, we cannot fail to express our wonder at the many marvellous things which appear to take place in Deutsch-land, and seldom or never in other countries. That faculty of second sense, by which they can often see and hear, when the perceptions of other mortals are laid asleep, is the offspring, we suppose, of their over-active imaginations, or sometimes, perhaps, of that intellectual dreaminess which many experience, when "primed" with the fumes of the Virginia weed; it seems to be an ignis fatuus, which delights to lead them astray from the high road of common observation, and of plain, obvious deduction, into the fens and quagmires of conjecture and of fantastic deception. None can be more willing to bear testimony to the high merits and surpassing excellencies of the Germans, in many respects, than we are; for where do we find such unconquerable perseverance, such unwearied industry, and so ardent a love of science, for science', and not for fortune's sake, as among them? It would seem that Nature is jealous of excessive kindness in the distribution of her intellectual favours, and that it is only on a very scanty few that she bestows the aggregate of them all. There is, as it were, a compensation-system adopted in the general award, so that, where one faculty or set of faculties is developed in an unusual degree, the superiority arising from their excellence is counteracted by the deficiency, or irregularity, of some other faculty or faculties; for example; compare and contrast the mind of a Frenchman with that of a German—what a striking difference! the quickness, alacrity, and light-hearted promptitude, the hop-skipping, grasshopper-like restlessness, the past-forgetting, novelty-attracting, and consistency-despising character of the one; and the patient, plodding, laborious hardihood of the other, with his fact-collecting acquisitiveness, burden-oppressed memory, and slow, self-bewildering judgment, oft led astray by his active, shaping ideality, just as a traveller is by the twinkling of a lanthorn-fly in a dark night.

Both of these characters have their advantages and evils; be it our part to cull the former and reject the latter. Few can have greater occasion to be impressed with the importance of this, than those engaged to cater for the English reader from foreign medical literature. Whether our Continental brethren are led to form as unfavourable an opinion of the merits of the British medical journals, as we reluctantly are compelled to form of theirs, we cannot tell; but, with all kindliness of feeling for them, and with an entire freedom from national partiality and prejudice, we hesitate not to affirm, that there is more practical information, more substantial food for the mind, more, in short, of strong, shrewd, every-day good sense in one number of any of our established periodicals, than in the annual series of most of the Continental.

But here we must now stop the vagaries of our pen, and resume our gleaning labours; and, as some may be of opinion that we have been harsh and hasty in our judgment, it may be as well to "shew cause" for it. This is no very difficult task. Perhaps the following report of a case of fatal hydrophobia, from the bite of a healthy and sound dog will satisfy our opponents.

A young countryman was taken suddenly ill; he complained much of a difficulty of swallowing liquids: no other distinct morbid symptom was present. Towards evening he had "un veritable acces de rage;" for observing his sister drink a glass of water, he fell into a violent passion, smashed a looking-glass near him, and besought his attendants to go away, or else he would bite them! This paroxysm lasted for about half an hour, when he fell asleep, and dozed quietly for two or three hours. At ten o'clock he had a second and similar attack; he began to cry, and bark like a dog, and had such an irrepressible desire to be thoroughly "canine," that he ran after his aged mother, threw her down on the ground and bit her cheek most severely; and not content with this enormity, he attempted to treat his sisters in the same way; but they (being more alert of limb, we suppose) made their escape from him; he took his revenge, therefore, on the furniture of the room, and broke to pieces every thing which he could lay his hands on. When this "acte de fureur" subsided, he became more rational, and when his family revisited him in the course of half an hour afterwards, they found him dead, "la tete cacheé dans les draps." It will no doubt be a satisfaction to the reader to be told, that "sa mere n'eprouva aucun accident par suite de sa morsure."

---

SUCCESSFUL LITHOTOMY IN ITALY.

In the course of last Autumn twenty-two cases of the lateral operation were performed by Professor Petrunti, in the Hospital of Incurables, at Naples: of these, nineteen were successful: eight occurred in children under five years of age; nine, from five to fifteen; two, from fifteen to twenty-five; and three, at later periods of life. The three unfortunate cases occurred in men, of 44, 56, and 48 years of age: in the first of these, convulsive symptoms made their appearance soon after the operation, and when they were subdued, a fatal peritonitis followed: the man died on the 14th day. In the second case, the calculus was found to be so large, that the dilatation of the internal wound was necessary; he died on the eighth day, from cystitis. On dissection the neck of the bladder was discovered to be lacerated, and in a sloughy state. The third was similar to the preceding one: the calculus weighed five ounces and two drachms: death from cystitis took place on the eighth day. On dissection, the left kidney was found to be hypertrophied, soft, and beginning to suppurate, and there was moreover an extensive deposition of purulent matter in the right iliac region.—*Annali Universali di Medicina.*

---

CASES OF LITHOTOMY AND LITHOTRITY, IN ILLUSTRATION OF M. BLANDIN'S MEMOIR, Reviewed in the present Number.

CASE 1. *Lateral Operation—alarming Hæmorrhage—Ligature of the Internal Pudic—Death.*

The lateral operation was performed after the usual manner, by M. Roux, in the year 1822, and the calculus was readily extracted; but a continual oozing of blood from the wound, after the patient was removed to bed, began speedily to exhaust his strength: the plug was first used, but this was soon found to be of no avail; and as the bleeding orifice was so deep that it could not be seen, M. Roux determined to tie the internal pudic in its course along the edge of the tuberosity of the os ischii. By this bold step, the hæmorrhage was immediately arrested; but symptoms of peritonitis soon afterwards set in, and the patient died. On dissection it was ascertained that the internal pudic itself near the bulb of the urethra had been wounded in the operation; it was situated much nearer the anus than is usually the case.

Case 2. *High Operation—Extravasation of Urine—Death.*

M. Amussat alludes to the case of a child on whom he had performed the high operation of lithotomy, to extract a calculus which was of an unusually large size, and which was found to be lodged partly within the vesical orifice of the urethra. On the third day, symptoms of a feverish exhaustion set in, and they became gradually more and more alarming. M. A. knew well the irremediable mischief that had taken place. In two days the patient died.

On dissection a most extensive infiltration of urine was discovered to have taken place; for not only was the subperitoneal cellular tissue in the iliac and lumbar regions filled with the fluid, but it had extended upwards as high as the diaphragm.

Case 3. *Lateral Operation—Vesical Phlebitis—Death.*

A child, three years of age, was lately cut for the stone in the Hospital Beaujon: the calculus was extracted with great ease, and for the next two days every thing went on most favourably; on the third day, however, shiverings, alternating with heats and sweating, and attended with a rapid pulse, great debility, &c. set in. There was but little pain in the hypogastrium, the urine flowed freely from the wound, and the edges of this seemed quite healthy. These unfavourable symptoms resisted every method of treatment; the shiverings became more and more frequent, the abdomen tympanitic, the stomach vexed with distressing vomiting, and the vital powers extremely languid: he died on the eighth day.

*Dissection.* The extremity of the bulb had been wounded in the operation; the internal incision had not divided the fascia of the prostate, and none of the large prostatic veins had been opened. The spongy tissue of the bulb contained some fetid purulent matter, and this might be traced along the veins which join the vena dorsalis penis, into the vein itself as far as the symphisis pubis; pus was found also in the superior veins of the prostate gland, and had become infiltrated into the adjacent cellular texture. The parietes of all the affected veins were thickened, opaque, and had their own capillaries or vasa vasorum highly injected. On examining the thorax, nearly a pound of semi-purulent effusion was found within the pleuræ; part of the right lung exhibited numerous patches of lobular engorgement; and in several of these patches, distinct purulent infiltration could be observed, while in others, the structure of the lung was only hepatised; a change, which is well known to be antecedent to the former.

Case 4. *Bilateral Operation—great difficulty in the Extraction—Recovery.*

A young man, 26 years of age, was operated on at the Hôpital Beaujon, in 1828, by M. Blandin, who adopted the bilateral method; the calculus was of considerable size, and of a very irregular shape. So much difficulty was experienced in extracting it, that the prostate gland was dragged fairly out to the outer wound, whenever an attempt was made to withdraw the forceps, and M. B. was obliged to order his assistant to push the gland up, while he continued to make the necessary efforts at extraction.

In spite of the "violence extreme excercée sur la prostate," this patient recovered, without having one unfavourable symptom.

Case 5. *Lithotomy and Lithotrity—Extraordinary tendency to the Formation of Calculi—Five Operations within Six Years.*

M. Poterlet was, in the year 1828, relieved of a calculus by means of lithotrity, performed by MM. Heurteloup and Amussat. No trace of any calculus could be detected after this operation. In the course of two years lithotrity was again performed successfully by M. Amussat. In the following year, another calculus had formed, and the patient himself gave the preference this time to the cutting operation, which was done by M. Amussat. In 1832, lithotrity was performed by MM. Heurteloup and Amussat for the third time; now again (Nov. 1834.) this patient has undergone two séances within the last week; the calculi consist chiefly of the phosphate of lime.

CASE 6. *Lithotomy—two Operations—Perineal Fistula with Calculus—death.*

J. Bertrand, 17 years of age, had undergone lithotomy twice, when admitted into the La Charité Hospital, in 1817, in consequence of a perineal fistula which had remained ever since the second operation. The urine which was thick, muddy, and fetid, escaped partly by the urethra and partly by the wound, and the patient was in a deplorable condition, from the constant dribbling. At times he suffered severe pain at the point of the penis; and after any exercise, it was not unfrequent, that blood was discharged along with the urine.

On introducing a sound along the urethra, M. Roux detected the presence of a calculus so firmly impacted within its vesical extremity, that it could not be dislodged. As it could be felt deep in the perineum, an incision was made directly upon it, but it was found too firmly fixed to be at once removed, and as by the finger introduced into the rectum, it was felt to be of considerable size, the incision was freely enlarged, so that the surface of the calculus was quite exposed: still it could not be seized with the forceps: at length by using the fingers instead of the instrument, the larger portion of the calculus was extracted; this portion consisted of two pieces, one of which was nearly as large as a pigeon's egg, and somewhat resembled in shape a flask, being contracted at one end, while the other, of smaller dimensions, was fitted to the larger end of the former piece, as the astragalus is to the os calcis; the portion which remained behind had been attached to the smaller of the two pieces described; when extracted with the forceps, it was found to be as large as a walnut. On the day following this severe operation, a quantity of purulent matter was discharged by the wound along with the urine: the patient gradually sunk, and he died on the 5th day. During a space of five years he had suffered from almost continual pain, from incontinence of urine, and from a suppurating sore, all of which causes must have seriously injured his constitution.

On dissection a quantity of sero-purulent fluid was found within the pleuræ; the mucous coat of the bladder, especially in the neighbourhood of the neck, was indurated and exhibited patches of ulceration.

CASE 1. *Lithotrity, with the Percuteur—Urethra at first extremely irritable—three séances necessary—Cure.*

Count L. aged 62, consulted M. Amussat in January, 1834, in consequence of a slight difficulty which he had experienced for some time past, in making water. On sounding the bladder, the presence of a calculus was immediately ascertained. The introduction of bougies and such like instruments caused at first very considerable irritation, attended with feverish symptoms, and so much swelling of the mucous membrane of the urethra, that M. Amussat was obliged to defer the operation of lithotrity for some time. By using warm baths, and taking mild opiates occasionally, the passage was brought into a less irritable state, and then the gentle introduction of instruments, gradually of a larger and larger size, persisted in daily for several weeks, enabled the patient to undergo the operation.

On the 8th of February, the first séance was submitted to; the instrument was passed into the bladder with facility, the stone seized and broken by the percuteur four different times: on withdrawing the instrument, it was loaded with a yellowish detritus: three or four moderately-sized pieces of the calculus were passed when the patient made water. The patient experienced a few slight pains during the operation which lasted altogether eight minutes. The second séance was held on the 12th; several fragments were successively seized and broken, and the patient experienced less pain on this than on the former occasion: the urine discharged immediately afterwards was not at all tinged with blood, as it had been before. Several fragments were discharged in the stream of the urine during the course of the night. On the 15th the operation was repeated: ten fragments were seized and broken: the sufferings of the patient were still less severe at this séance. For several subsequent days, a considerable quantity of the sandy detritus, and several pieces of calculus were discharged. On sounding the bladder then, M. Amussat satisfied himself that the patient was entirely freed of his disease.

Case 2.—*Lithotrity with the Percuteur—five séances necessary—Cure.*

A gentleman aged 46, who had suffered from symptoms of calculus since the year 1814, applied to M. Amussat, in Nov. 1833. The first introduction of a large sound was followed by a troublesome nervous fever, which continued for upwards of a week: the bladder and urethra had always been exceedingly irritable in this patient. At the first séance, the calculus was seized and broke into several fragments: no unpleasant symptom was induced; but after each of the three following operations the patient suffered exceedingly; a copious hæmorrhage on one occasion came on, and reduced his strength so much, that the treatment was necessarily protracted for a very considerable time; and what added to the difficulties in the present case, was, that the bladder seemed to have lost much of its expulsive energy, for but little detritus and scarcely any pieces of calculus were discharged with the urine. A fifth séance was necessary for the entire removal of every portion; and although for three or four days after this the patient experienced severe lumbar and vesical pain, he soon began to recover his strength, and in the course of a week or two, he found himself altogether better and more comfortable than he had been for the preceding twenty years; he could now walk and take considerable exercise even on horseback, without suffering any distress.

Case 3. *Lithotrity by means of the "Brise-pierre,"—one Séance—Cure.*

A middle-aged man was recommended by Professor Dubois to the care of M. Amussat, for the purpose of relieving him from a urinary complaint. This patient had a few days previously passed a calculus of the size of a cherry-pip: the canal was therefore in a favourable condition for the introduction of the necessary instruments: these were introduced for several days successively, in order to dilate the urethra still more. The operation with the "brise-pierre" was performed with great ease, and with so little distress to the patient, that he had believed that M. Amussat was merely sounding his bladder. "If," says the narrator, "we had to do only with such cases, lithotrity would scarcely deserve to be styled an operation."

Case 4. *Lithotrity—Inflammation of the Bladder—Death.*

M. P. applied to M. ——, in consequence of suffering much from a urinary complaint. A stone was detected on sounding the bladder, and he was recommended to have the operation of lithotrity performed. Several attempts were made by the surgeon to lay hold of the calculus with the "brise-pierre," but these were unsuccessful, and unfortunately they could not be again repeated, for symptoms of cystitis came on, and in 15 days the poor patient was dead.

[It is to be regretted that the details of this case are so meagre; it is not even stated whether the operation was performed lately or several years ago, when it was still imperfect and faulty.]

To the preceding case we may add, that in one instance, reported in Baron Larrey's Report on M. Civiale's cases, the patient, "tomba dans les accidens nerveux si graves," after the first séance of lithotrity, that he died on the third day subsequently.

Case 5. *Lithotrity—the attempt unsuccessful—Death on the fourth day.*

A young man, 17 years of age, was admitted into the Hospital Beaujon in 1828, with a calculus in the bladder. M. Blandin made an attempt to seize it with the forceps "à trois branches," but he was unsuccessful: in the course of a few days, a second equally fruitless attempt was made; and on this occasion the patient suffered considerably more pain. On the following day the patient experienced pain in the renal and in the hypogastric regions, frequent desire to make urine, &c.; these symptoms were attended with general pyrexial irritation. The pain and fever increased, the scrotum became retracted, and almost continual retching and vomiting supervened. He died on the evening of the fourth day.

*Dissection.* On examining the bladder, its mucous surface, especially of the cervix, was very highly injected; the ureters were dilated, and exhibited a livid red colour on their inner surface; the urethra was perfectly sound; the kidneys were larger than natural, and indicated a tendency to ramollissement of their structure.

The calculus within the bladder was of the size of a walnut.

Case 6. *Lithotrity forbidden by excessive Irritability of the Urinary passages.*

M. Marmot, 60 years of age, had laboured under the symptoms of calculus for three years, when he applied to M. Blandin; the bladder had always been excessively irritable, and the constitution of the patient was highly nervous: indeed so alarmed had he been about his malady, that he would not submit for a long time to being sounded; and when he did, the examination was so unsatisfactory that M. B. could not ascertain any thing beyond the mere presence of a stone; he could not form any idea of its size, of the capacity of the bladder, and so forth. Several subsequent attempts were made to accustom the urethra and bladder to the contact of instruments; but these were all fruitless; and indeed on one occasion, disagreeable feverish symptoms, and very considerable strangury was induced; and the patient continued in this unpleasant state for several months; all idea therefore of any operation was abandoned, and M. M. still retains his malady.

Case 7. *Lithotrity—Abscess in the Prostate—Six Séances necessary—Cure.*

M. D., 71 years of age, after having suffered from calculous symptoms, consulted M. Blandin in 1829. At the first séance the calculus was broken into several pieces; the bladder was so strongly contracted that it would not receive more than an ounce of injection, and even this small quantity was almost immediately rejected. After the second séance, when considerable difficulty was experienced in introducing the straight forceps, there occurred a retention of urine, occasioned by an abscess having formed in the prostate gland. The introduction of a catheter to draw off the urine, was attended with extreme difficulty; but at length a small sound was passed. A month was allowed to elapse, before any further attempt was made on the calculus. The progress of the case was very tedious, as the bladder had not the power of expelling its contents, and the debris of the calculus was therefore voided only through the catheter; and this instrument required to be used during the whole time. After six séances (in all) no traces of a calculus could be detected. In half a year after this date, M. D. again experienced all his former symptoms, and it was then ascertained that another calculus had been formed. He submitted to a "nouvelle application du broiement, et l'extraction artificielle de fragmens de pierre," which was much less hard than the former calculus; the one being composed of the ammoniaco-magnesian phosphate, and the other of uric acid. It is two years since the date of the last operation of lithotrity, and the retention of the urine still continues. The bladder seems to have become paralytic.

Case 8. *Lithotrity—Three Séances. Attempt ineffectual—Lithotomy—Death.*

M. Rousseau having long laboured under great distress from urinary calculus, consulted M. Civiale.

At the first séance the stone was seized, but not without some difficulty, and submitted to the operation of the "foret;" the three following séances were "tout a fait infructueuses," for the calculus could never be fairly embraced by the forceps. The patient now applied to M. Leroy, who found on examination that the calculus was about twenty lines in its longest diameter, while its antero-posterior diameter was comparatively very small; that the bladder was hypertrophied and excessively contracted; that the prostate was greatly swollen, and that the neck of the bladder formed a sort of long and narrow "vestibule."

These circumstances determined M. L. to decline performing any lithotritic operation; but the patient insisting upon one attempt more being made, he was recommended to use opiate injections into the bladder for some time before any instruments were introduced. Unfortunately this treatment seemed to produce but little benefit, and M. Leroy was therefore obliged to hazard the operation much against his own will. The bladder was found to be so very irritable, that it would not retain above an ounce of fluid, and even the extremity of the catheter was so firmly grasped by the contracting viscus, that "on ne pouvait lui imprimer aucun mouvement:" M. L. waited some time, with the view of giving time to the spasm to relax, as it usually does; but it, as well as the pain, rather increased than otherwise; he therefore closed the branches of the instrument with the greatest possible caution, to avoid injuring the coats of the bladder, and slowly withdrew it. A large dose of opium was administered, and the patient kept quiet for several days. At the following séance M. Heurteloup assisted. He recommended a trial of the forceps "a quatre branches mobiles independantes," or, as it has been called, "evideur à forceps;" but the stone could not be seized, for it was retained in the "bas fond" of the bladder by the prostate, and therfore lay below the reach of the instrument. Neither injections, nor the "pince servante," nor "la bascule" of the rectangular bed were of any avail in displacing the calculus from its position. Another trial was made a few days afterwards, and on this occasion M. Heurteloup succeeded in grasping the calculus, but by no means advantageously, for it was not sufficiently embraced by the branches of the forceps, to permit the "evideur" to act with much power. At a third séance, but very little progress was made, and the patient on this, as indeed on the two former occasions, suffered extreme pain.

It was now decided that lithotrity must be abandoned, and the lateral operation of lithotomy was performed by M. Hervez de Chegoin. When the internal incision was made, a stream of purulent matter flowed out of the wound, and it was found that this had escaped from an abscess in the prostate, which had recently formed, and had caused extreme suffering for several days to the patient. Unfortunately, the pain did not cease after the extraction of the calculus, but continued with great severity, until the death of this patient, who sank exhausted on the twelfth day.

On dissection, the prostate was found to have attained the size of an orange; the parietes of the bladder to be nearly a quarter of an inch thick, and its cervix to be at least an inch and a half in length. The calculus was of a long ovoid form, and exhibited one perforation "legerement excavée." We may therefore suppose that the "foret" of M. Heurteloup had entered the pit which had been previously made by M. Civiale.

Case 9. *Lithotrity—Three Séances—Impaction of a Fragment in the Urethra—Death.*

A man, about thirty years of age, had, under the influence of one of those strange mental aberrations of which the genital organs are often alike the cause and the object, introduced the stalk of a long grass into his urethra;—the end of it having broken off, slipped into the bladder, and there had become the nucleus of a stone. The patient entered the Hôtel Dieu, and M. M. Dupuytren and Breschet, considering the case as well suited for lithotrity, entrusted it to M. Blandin.

The sound detected the presence of several calculi in the bladder; two of them were readily seized and crushed with the forceps and drill; a third, being much harder, required the use of the bow. While M. B. was using this, the patient on a sudden made a violent struggle, and shrunk back so much from the instrument, that its extremity was drawn within the urethra. Fortunately, by this time, the calculus had been crushed, and the blades were already closed, else the neck of the bladder must inevitably have been lacerated. M. Blandin candidly confesses that he had omitted to secure the body of the patient with the shoulder-straps.

Two other séances were successively undergone, and several calculi crushed and discharged: but unluckily a fragment of one larger than the rest became impacted in the membranous portion of the urethra, and could not be dislodged; and although it did not much obstruct the passage of the urine, it prevented the introduction of any instrument into the bladder. Things continued in this state for several days, when the patient, upon leaving a warm-bath, caught cold, and was seized with a violent pneumonia, which speedily proved fatal. On dissection, the calculus was found in the membranous portion of the urethra, which, as well as the prostatic portion, had become much inflamed. The adjacent veins were found to contain pus. Numerous small abscesses were disseminated through the substance of both lungs. A score, at least, of small calculi were found in the bladder.

[This case we have already reported in our review of M. Blandin's Essay. We have thought it right to reproduce it here with the other cases, in order that the catalogue may be more complete, and thus furnish at a coup d'œil examples of the most frequent and important dangers of lithotrity.—*Rev.*]

CASE 10. *Lithotrity unsuccessful—Recto-vesical fistula—Lithotomy—Cure.*

M. Turgot, who had undergone several séances, but in whom the calculus could not be effectually removed by lithotrity, applied to M. Dupuytren, who advised him to be cut. The operation was performed, and this patient was relieved not only of the calculus, but also of a recto-vesical fistula: (the origin and date of the fistula are not stated.)

CASE 11. *Lithotrity—End of one of the Instruments broken off in the Bladder—Twenty Séances—Cure.*

M. F., aged 72, had laboured under symptoms of stone in the bladder for a period of five years, when he applied to M. Heurteloup. The Baron operated with the forceps "à trois branches droites" four different times. Being then called away to London, he entrusted his patient to M. Leroy's care, who discovered in the bladder a number of soft calculi, the detritus of which, when deposited, resembled mortar.—This species of calculus very generally indicates an unhealthy condition of the urinary passages, and in this patient there existed an incomplete retention of urine. The case was therefore by no means a favorable one. After every séance it was found necessary to discharge the fragments, and debris of the calculi by means of injections, and of the "sonde evacuatrice." Several pieces which were detained "dans les yeux de cette sonde" were crushed, and then voided in powder. Twenty séances in all were undergone by M. F. before he was declared cured of his malady. He also got rid of the strangury at the same time. An accident occurred at one of the séances, which deserves to be mentioned. While M. Leroy was in the act of drawing back the "foret" or perforator within the blades of the forceps, its extremity broke off, and was left behind in the bladder. The patient was quite ignorant of what had taken place, and was so little discomposed by the séance, that he walked home without any distress. M. Leroy hoped that the fragment might become admitted in the apertures of the "sonde evacuatrice" and be removed in this way; but not so. Several times it was laid hold of with the forceps, and it was easy to distinguish it from the calculus by the sound which it gave out when struck with the perforator. When nearly all the calculi were got rid of, M. Leroy was then fortunate enough to extract the broken-off end of the instrument by means of the ordinary forceps " a trois branches."

We may add that M. Hervez de Chegoin lately extracted by lithotomy a portion of a litholabe, which had been broken off by accident in the bladder.

Case 12. *Lithotrity — Thirty Séances — Cure.*

In 1829, M. Leroy was consulted by an officer, with the view of having a urinary calculus removed by lithotrity. The calculus was a large one, measuring upwards of two inches in its long diameter, and it was rough and irregular on its surface. The patient was informed that the treatment would necessarily be very tedious. The bladder being easily dilatable, no difficulty was experienced in seizing the calculus, and attacking it with the "foret à ailes." The progress which was made at this and at a second séance was very little; and M. Leroy, therefore, determined to use " les ailes du foret" to break the calculus in pieces. Some of the fragments were then reduced " par eclatement," and others were crushed.— Thirty séances in all were necessary, before every fragment was quite removed.

Case 13. *Lithotrity—Ten Séances —Cure.*

M. Alexis was a patient of M. Leroy. The calculus was ascertained to measure rather more than an inch and a half lengthways. The bladder was in a favorable condition. At the first séance the calculus was broken in pieces, "par eclatement," after having been perforated. By other nine séances the bladder was freed from any vestige of a fragment, and the patient restored to perfect health.

Case 14. *Lithotrity impracticable, in consequence of the flattened Shape of the Calculus.*

A gentleman who had for many years suffered great distress from a urinary calculus, but who was unwilling to submit to any cutting operation, applied to M. Leroy to have lithotrity performed. M. L. found on examination that the calculus was of a large size, and moreover that it was so flattened in its shape, that it repeatedly slipped from the forceps, after being seized:—he therefore advised his patient to submit to lithotomy. The stone was extracted without difficulty, but the patient died on the third day.

Case 15.—*Lithotrity impracticable, in consequence of the flattened shape of the Calculus—Lithotomy—Death.*

A gentleman, 40 years of age, applied, in 1830, to M. Leroy for relief from a urinary calculus, which had distressed him for many years. The bladder was hypertrophied, and could not hold much more than a wine-glassful of fluid. The desire to pass water was therefore most troublesomely frequent; the prostate gland was rather enlarged; the calculus was pronounced to be of a flattened shape, and to measure lengthways about 16 or 18 lines. The circumstances of the case were not favorable for the success of lithotrity, nevertheless it was attempted.

At the first séance the stone was seized with the forceps " a trois branches" and then attacked with the perforator, provided with " ailes articulées," so as to craunch it down from before backwards; but as the calculus presented its thin edge, on which the " ailes" caught, and the perforator could not move easily, M. Leroy therefore contented himself with making a simple perforation at this séance. The attempt was renewed a few days afterwards, but the bladder was found to be so exceedingly irritable, that no instrument could with safety be moved about in the bladder. The dysuria was aggravated in consequence for upwards of a week. Lithotrity was therefore abandoned as scarcely practicable, and the patient was cut by M. Bouchet, who extracted two flattened calculi. Death happened on the 8th day.

Case 16. *Lithotrity successful on two occasions.*

M. G. aged 70, applied in 1829 to M. Lisfranc, who detected the presence of a calculus in the bladder. This patient had for many years been subject to attacks of retention of urine, and this secretion was usually turbid, and loaded with mucosity.

In spite of these unfavourable circumstances, it was determined by MM. Lisfranc and Leroy to attempt the destruction of the stone by "le broiement" with the forceps "a trois branches, munie d'un foret à developpement." The calculus being rather soft and friable, was entirely removed at the ninth séance. Six months after this date, another calculus had formed; the patient, in the mean time, having enjoyed very good health, with the exception of the retention of urine, which had so long troubled him.

M. Leroy repeated his lithotritic operations, and again freed M. G. from all traces of a calculus, which, it deserves to be noticed, belonged to the ammoniaco-magnesia phosphate class.

Case 17. *Lithotrity in a Girl, three years of age.*

This child had suffered from the symptoms of urinary calculus for twenty months. She had been repeatedly sounded by different surgeons, but no calculus had been found.

M. Segalas, however, was at length so fortunate as to satisfy himself of its presence in the bladder, and he determined to attempt its removal by lithotrity.

Six séances were necessary for its entire destruction and discharge.

M. Civiale relates a case of successful lithotrity which he performed on a boy 13 years of age.

---

### On Hæmorrhoids and Prolapsus of the Rectum.

Case 1. *Severe Pains experienced only during Coition—Disease of the Uterus suspected—Cure.*

It is by no means an unfrequent occurrence in hospital practice to find women who are labouring under hæmorrhoidal affections, and who are ignorant all the while that these very affections are merely secondary, and are dependent upon some disease of the uterus, or of the vagina. On the other hand, experienced surgeons are well aware, that a uterine or vaginal disease may be most perplexingly simulated by internal hæmorrhoids, as is proved by the following case:—

A woman, 23 years of age, complained that she had always suffered severe pain during coition. The husband, finding that month after month passed away, and yet that things were nowise better, became discontented and suspicious of the affections of his wife; he applied therefore to a midwife to examine her; but she could not discover any cause why the lady should be more unfortunate than others of her sex. In consequence of this a physician was applied to, and when he was informed that the pains were experienced only during connexion, he proposed a manual examination, and thus ascertained, that through the posterior wall of the vagina several tuberculous masses might be felt. The patient now confessed that for a length of time she had occasionally experienced annoyance at stool, and that blood was sometimes discharged with the evacuations: that this hæmorrhage usually afforded her considerable relief, so that she could then permit her husband's approaches with less suffering.

By appropriate treatment, directed chiefly to the relief of the rectum, this woman got rid of all her distress, and soon afterwards became pregnant.

Case 2. *Disease of the Rectum mistaken for Scirrhus of the Uterus.*

Mad. G. had been always regular until she reached the age of 44, at which time a tedious hæmorrhoidal flux supervened, and the catamenia gradually ceased some months afterwards. This lady began to experience a sense of weight and uneasiness in the perineum, and then sharp and lancinating pains within the vagina, shooting up to the region of the uterus. The physicians first consulted considered the case as one of incipient carcinoma of the uterus; but as no amendment was afforded, M. Lepelletier was applied to. Upon carefully examining per vaginam, he could not trace any sign of disease of the uterus, and the only lesion which he could detect was an induration at the superior and posterior wall of the vagina. He now examined the rectum, and discovered, high up, that there were several painful, ulcerated, hæmorrhoidal swellings, and also that this surface of the recto-vaginal septum had become thickened and scirrhous. Only temporary relief could be promised in such a case; the patient died in 15 months after M. L. first attended her.

Case 3.—*Influence of the Catamenial and Hæmorrhoidal Fluxes in Phthisis Pulmonalis.*

A young lady had, previous to the first appearance of the catamenia, all the symptoms of approaching phthisis pulmonalis, and even although these symptoms almost entirely ceased after menstruation had become established, her physician regarded her as in a very precarious state, for the lungs were, in his opinion, decidedly tuberculated. The lady, however, enjoyed very tolerable health up to her forty-fifth year, at which period the catamenial flow began to cease, and her pulmonary complaints to reappear. Fortunately, a vicarious hæmorrhage from the rectum supervened, and again the chest affection abated; the hæmorrhoidal discharge continued (periodically, we suppose) year after year, till the patient attained her 65th year—it then ceased, and soon afterwards she died of confirmed phthisis.

Case 4.—*Prolapsus of the Rectum, treated with the Actual Cautery.*

A young man, 22 years of age, had laboured under a prolapsus of the rectum for upwards of a year; it gave him great pain, and induced a most distressing tenesmus, and not unfrequently troublesome hæmorrhage. The gut could be reduced, but not retained when up.

A variety of means had been tried in vain; recourse was, therefore made to the actual cautery. It was applied once a week to the whole surface of the exposed gut; the swelling gradually abated, and the hæmorrhage and tenesmus became less and less troublesome. The intestine shrunk up in course of time, the ulcerated surface healed, and the patient was entirely cured of his very distressing malady in about two months from the first application of the cautery. No return of the disease took place subsequently.

Case 5.—*Prolapsus of the Rectum, treated with Caustic Ligatures.*

A man, 72 years of age, had for several years laboured under a prolapsus of the rectum which had become as large and bulky as the worst case of prolapsed uterus. Boyer, whose patient he was, dreaded an alarming hæmorrhage from excising the protruded gut, and he determined, therefore, to surround its base with a ligature of several cotton threads, previously wetted with caustic potass. On the morrow, he made an incision through the circular eschar thus produced, and then applied a ligature of several silk threads, well waxed, and tightened it more and more for several days successively. Considerable constitutional disturbance ensued. On the fourth day, the ligatures were found to have worked their way to nearly an inch in depth through the substance of the prolapsed mass; Boyer, therefore, with one sweep of the scalpel excised it all. Several arteries required ligatures, and a plug was likewise introduced into the rectum, to keep up a steady pressure for some time.

Case 6.—*Prolapsus cured by the Excision of several Folds of the Integuments round the Anus.*

A young and healthy woman was admitted into the Hôtel Dieu, for relief from a troublesome protrusion of the rectum; it was not complicated with any hæmorrhoidal affection, nor, indeed, was it always induced by the usual cause—straining at stool; for sometimes she would not be annoyed with it for two and three weeks, and then it would recur without any very apparent cause. But this occasional distress, and the almost constant discharge of a bloody mucus from the anus, were grievances sufficient to induce her to submit to an operation. M. Dupuytren excised four folds of the integument without, and of the mucous coat within, the anus, and applied simple dressings to the wounds. In a few days, this patient was completely relieved of her malady.

The preceding six cases are extracted from M. Lepelletier's Concours Essay.

CASE 7.—*Prolapsus of the Rectum cured by the Application of the Actual Cautery.*

A. M. aged 50, was admitted into the hospital at Gand, in consequence of an old and troublesome prolapsus. It was as large as a large apple, and its mucous surface was swollen, irregular, hard, and almost callous.

Three cauteries, heated to whiteness, were successively applied to the mass, and Professor Kluyskens, not satisfied with this, introduced one of them into the anal orifice, so as fairly to touch every portion of the protruded gut, and reduce it to an eschar; simple dressing was then applied, and kept it in its place by a T bandage. The pain produced by the cauteries was only temporary: the bowels were kept gently open, and the patient recovered without having experienced one unfavourable symptom. When he left the hospital, there was not the slightest tendency to prolapsus.

The operation, in the preceding case, may be deemed by some surgeons to have been too severe, and they may suppose that Dupuytren's mode of excising three or four folds of the integuments round the anus, might have succeeded perfectly; but let them remember, that the protruded mass was much changed from its normal structure, and even if it could have been kept up within the sphincter, this was not to be desired. We admit that Dupuytren's operation is sufficient for most recent cases. For many years, Prof. Kluyskens has been in the habit of using the actual cautery to prolapsus of the rectum, and almost invariably with perfect success; the pain is by no means so severe as might be imagined, and the danger of consecutive accidents may be averted by judicious treatment.—*L'Observateur Medical Belge.*

---

### SOLANINE IN POTATOES—POISONING FROM MUSHROOMS.

Several accidents among the cattle which had been fed with the residue of the potatoes (in a state of germination) used in the preparation of brandy, having occurred lately at Brunswick, M. Otto was induced to perform some experiments.

He treated the germs with water, slightly acidulated with sulphuric acid —then added the acetate of lead to precipitate the acid, and filtered the liquor; to this liquor lime was added, and the precipitate thus obtained was treated with boiling alcohol, which dissolves the solanine; the alcaloid may then be purified by repeated solutions and crystallizations, One grain of the solanine killed a rabbit in six hours—and three grains a dog in nine hours.

In the same periodical, we observe the history of four individuals having died from eating mushrooms. The species of agaricus which had been eaten were ascertained by M. Brogniart to be a. grammapodius—a. infundibuliformis —a. fusipes, and a. pectinaceus of Bulliard (Emeticus, of Persoon, and Integer, of Sowerby.)

The three first species are usually considered innocuous; but the last is very poisonous. It is an interesting problem in organic chemistry, to discover the noxious principle of the agarici; hitherto, it has escaped the tests of the analyst.—*Journal de Chimie.*

---

### ON CUTANEOUS DISEASES.

In our last No. we gave an ample analysis of M. Rayer's treatise on the diseases of the skin, and we then commented freely on what we considered to be the errors of his arrangement, and the defects of the French school in general, on this subject of medical inquiry. Our readers will find that we gave the preference to the standard works of our own countrymen, Drs. Willan and Bateman, not only because they are of a more practical character, but because the arrangement adopted, though imperfect, and not unfrequently faulty, is less hypothetical, and of more easy application. It is always gratifying to us to find our opinions on foreign authors coinciding with, and, therefore, confirmed by, the judgment of others, and more especially of the author's own countrymen; such is the case in the present instance. M. Gibert, educated for many years under M. Biett, one of the ablest dermatologists of France, and now "Professor of Cutaneous Pathology" at Paris, has recently published a manual on skin diseases, wherein he candidly and unhesitatingly decides in favour of Willan's system, over any one proposed subsequently, either in France or elsewhere. He makes eight orders, according to the appearances and external characters of the eruptions; he very properly (from a manual) excludes from each order those diseases which have always been considered to belong to the domain of general pathology, such as erysipelas, and also those which are usually treated of in separate works, or monographs, as variola, vaccinia, &c. and, in place of these, he has appended to each order the cognate or correlative eruption, induced by the syphilitic poison; for it is well known that the cutaneous diseases arising from this prolific source, exhibit almost every variety of cutaneous exanthema, retaining, however, some common characters and family resemblances, which indicate their parentage.

It is, therefore, not without good reason, that M. Gibert has devoted a chapter, at the close of his work, to a general resumé and grouping together of the different syphilitic eruptions, whose special and particular characters have been described in their proper places.

As a specimen of M. Gibert's treatise, we shall extract a sentence or two.

"In the English arrangement, the term 'lepra' has a meaning very different from that which is affixed to it by medical men in other countries: it is not applied to that dreadful and hideous disease, which is often fatal, and almost always incurable, but to a mere scaly eruption, which, in most cases, is of easy removal; the former belongs properly to the order 'tubercula,' and is the 'lèpre tuberculeuse' of Alibert, but we shall describe it under its true name, elephantiasis of the Greeks—whereas the latter belongs to the 'maladies dartreuses ordinaires' (would that the 'word' dartre were banished, as an outcast from medicine), and is the herpes furfuraceus circinatus of Alibert. For our part, we have no hesitation in adopting the sentiments of the English physician in preference to those of our own countrymen; and we are of opinion, that the terms 'psoriasis' and 'lepra' should be restricted to cutaneous affections of a scaly or squamous character; indeed these two diseases ought not, perhaps, to be separated at all, for they differ from each other only in the shape of the patches, and not in any essential particulars."—*Manuel des Maladies Spéciales de la Peau. Par C. M. Gibert. Paris,* 1834.

---

### Endermic Use of Digitalis in Dropsy.

The narrator of the following cases assures the profession, that he has been in the habit of employing digitalis, in the endermic method, for the last 20 years, with the most gratifying success.

Case 1.—*Anasarca and Ascites—Frictions on the Abdomen with the fresh Herb—Cure.*

A countryman became the subject of dropsical effusion into the abdomen and subcutaneous cellular membrane, on the disappearance of a "dartrous" affection of the skin with which he had been troubled for some time. The usual diuretic remedies having been tried without effect, the physician had recommended that the limbs should be scarified, and that paracentesis abdominis should be performed; but fortunately, having heard of the good effects of digitalis, used endermically, he resolved to make a trial of the remedy. The fresh herb was well beaten, and blended with some gastric juice, and this mixture was rubbed powerfully on the abdomen. By the fourth friction, the urine had begun to flow so very abundantly, that the patient, fatigued with rising from bed every minute, " prit le parti de s'asseoir sur le bord du lit, de mettre les pieds sur des chaises, une chaudron à terre." In the course of one night, the abdomen had greatly diminished in size; the diuresis was kept up, though not to the same extent, for several days, and, within 10 days, the patient was completely relieved of every dropsical symptom.

Case 2.—*Anasarca after Scarlatina—Frictions with the Tincture—Cure.*

A child, five years of age, while recovering from an attack of scarlatina, became affected with ascites. Frictions were ordered to be made on the abdomen, and inside of the thighs, three times a day, with half an ounce of the tincture of digitalis. In three days, the flow of urine had greatly increased, and the dropsical effusion was very visibly diminished; the frictions were then used only twice a day, and this treatment continued for about a week—the cure was complete.

Case 3.—*Ascites—Frictions with the Tincture—Cure.*

A labouring man, much addicted to the abuse of spirits, consulted M. Chretien in consequence of dropsy of the belly, under which he had laboured for some time. No internal medicines were prescribed, and the only remedy was the rubbing in, three times a day, of half an ounce of the tincture of digitalis on the abdomen, and on the inside of the thighs. At the same time, the patient was recommended to abandon the use of ardent spirits. The quantity of the tincture was gradually increased to two ounces per diem. The abdominal effusion very speedily began to diminish, and at the end of a fortnight, was quite absorbed.

Twenty ounces, in all, of the tincture were used.

Case 4.—*General Dropsy—Frictions with the Tincture, in Conjunction with Internal Remedies—Cure.*

Dr. Chretien was called to a man, whom he found in so advanced a state of general dropsy, that he considered that it would not be safe to have recourse at once to the digitalis. He ordered a stomachic strengthening wine, and small doses of subcarbonate of iron, with infusion of rhubarb (a combination which, the Doctor tells us, he very highly recommends in debilitated states of the system.) In the present case, however, no benefit was derived from its use, and frictions with the tincture of digitalis were accordingly commenced, in conjunction with the above internal remedies. In the course of a few days, the alvine and urinary secretions were much increased—the breathing, which had hitherto been greatly distressed, was easier, and the distention of the abdomen was very sensibly diminished; the anasarca of the extremities and of the scrotum was still very considerable.

In order to ascertain whether the cure would proceed without the internal means being adopted, they were discontinued for a few days; but, as the state of the patient became decidedly worse, recourse was again had to the infusion of rhubarb, with what the French call " safran aperitif de Mars." It required perseverance in the treatment which we have explained for nearly four months, to effect the perfect recovery of this patient.

Case 5.—*Ascites—internal and external Use of Digitalis—Cure.*

A woman, who had laboured for some years under cough and dyspnœa, became dropsical; the urinary secretion was remarkably deficient, scarcely more than a wine-glassful being discharged in 24 hours.

Pills, consisting of digitalis and of assafœtida, were ordered to be taken three times a day, and the tincture of digitalis was to be well rubbed in upon the abdomen. In the course of a few days after beginning the use of these remedies, the flow of the urine was so abundant, that the patient was obliged to get out of bed almost every hour; the general health became also, at the same time, much better, and, in the course of a month, every unpleasant symptom was entirely removed.

*Observations.* The tincture which was employed in the preceding cases was stronger than that in common use; it was prepared by digesting an ounce of the leaves of the herb in three ounces of alcohol. If the experience of other practitioners should confirm that of Dr. Chretien, the endermic method of exhibiting digitalis will be a most valuable improvement in the mode of treating many cases of dropsy. The reports of his cases are certainly far too brief, and too carelessly drawn out, to satisfy the mind of the critical reader. No allusion is made to the state of the pulse during the continuance of the remedy; but we are led to infer, from the subsequent remarks of the author, that it was always lowered, when administered internally, and that no sensible effect was produced upon it by the external use of the medicine. It has been maintained by Broussais, and other physicians of the physiological school, that the direct and normal action of digitalis is a sedative to the circulatory system, and that, whenever the pulse becomes quickened, or, indeed, is not reduced by its use, we may conclude that some gastric irritation exists in our patient; in order that the drug may be able to exert its natural action, the stomach must be in a tranquil state. The experiments which Dr. W. Hutchinson instituted upon himself are the most satisfactory and conclusive, as to the operation of digitalis on the human body, and they certainly confirm the doctrine of Broussais; the pulse was gradually more and more lowered as the medicine was continued, and its dose increased, until it beat only 28 times in the minute; and a gastro-intestinal irritation, accompanied with extreme exhaustion, having then come on, the contractions of the heart became strong and vigorous, and were upwards of a hundred. It is to be remembered, that this experiment was repeated, and the same results exactly were obtained as on the first occasion.—*Révue Médicale.*

---

### Strictures on the Broussaian Method of investigating Diseases.

The physiological, or, as it ought rather to be called, the pathological system of nosology, which has been so fashionable, especially in France, for the last five and twenty years, is, like all exclusive doctrines, beginning to lose somewhat of its ascendancy. Some, indeed, of the best physicians of Paris have, ever since its birth, been its inveterate enemies, and, clinging to the old order of things with all the pertinacity of devotees, have, through good and through bad report, exerted themselves to shew the extravagances of the modern anatomists. When we mention the names of Recamier, Bayle, Cayol, Gibert, and Martinet, as disciples of what they themselves call "the Hippocratic school of medicine," our readers will be satisfied that we have not misapplied the expression—"some of the best;" and, perhaps, we should not be far wrong, if we were to extend the list, and to enumerate among these the very man, who, perhaps, of all others, has exalted and promoted the science of pathological anatomy in the present century—we mean M. Andral; for, although no one has done so much, and written so well on the subject, yet it is very generally known that he is not "a physiological physician" in his treatment of diseases; he does not, like the Doctor of the Val de Grace, insist that every malady, to which our bodies are subject, is invariably associated with, if not absolutely dependent upon some lesion of tissue or structure which is, or ought to be, appreciable on dissection to the senses; or that this lesion is, in almost all cases, an inflammation, or something like it; and that, when not discoverable by dissection, we may be nevertheless assured, that it did exist during life, and has only been effaced during the agonies of death, or immediately afterwards. No; M. Andral is far too enlightened a philosopher to addict himself to any such absurdities, for well he knows that the animal body is too complicated a machine, and that it is acted upon by too many powers, both from within and from without, to be subjected to one lesion, or one set of lesions only. Of a kindred mind is M. Lobstein, the professor of pathological anatomy in the school of Strasbourg, and the author of a very able treatise on this subject. It has been the perusal of this treatise that has suggested the preceding remark; and when we find that those who write best on the all-absorbing topic of the so-called physiological physicians, and who yet do not adopt their dogmas in practice, a strong presumption is naturally suggested that, just in proportion as we become more and more intimately acquainted with the details of morbid anatomy, so do we become less and less confident in our explanations of the proximate causes of diseases. We have not made any allusion to that shining light of English pathology, the late Dr. Matthew Baillie, who is justly admitted by all to have been a consummate anatomist, and, withal, one of the most modest of nosologists: he looked at Nature as she presented herself to his view; he did not presume to dictate to her, nor yet to guess and conjecture at those mysteries which she has put beyond the ken of human faculties; but with the mind of a true philosopher, kept his way between the phantasies of the mere dreamers on the one hand, and the audacious arrogance of the dissecting materialists on the other.

But it is time to recur to our author. In one respect M. Lobstein differs most materially from most modern anatomo-pathologists, in attributing a mighty influence in the generation and developement of organic lesions to the nervous system, and to the varying states of the animal fluids. To some depravation or another of the functions of the one, or of the qualities of the other, many diseases, such as fluxions, congestions, inflammations, &c. are in their first workings attributable. M. Lobstein perhaps carries his ideas of the operation of the nervous system too far, and almost touches upon the domain of the old chemical philosophers, who were so apt to try to solve many of the enigmas of life by referring them to an archæus, or presiding animus. In one passage he has these words: "if in a physical science it were permitted to employ allegorical images, and to personify the conceptions of our minds, I should say that the nervous influence holds in subjection the universality of the humoral system, and rules it with an absolute power."

And again: "The hypothesis that there exists a peculiar principle, 'd'une matiere imponderable, elaborée par les nerfs, qui en sont en meme temps, les conducteurs,' seems to me very probable; and I suspect that this principle or agent is generated in the pulp of the brain, spinal marrow, nerves, and ganglia."

It is certainly of importance to distinguish well those symptoms of a disease which precede any appreciable alteration of texture, and which may be called "symptomata proegomena," from those which follow, and may be induced by these lesions, and which may therefore be called "symptomata epigenetica." By attending to this distinction, we shall possibly be enabled to discover the point where the disease ceases to be "dynamic," or as it has hitherto been improperly designated, "functional," and where the "organic" alteration or lesion commences.

In our author's opinion "a primitive disturbance of the vital powers invariably precedes all changes of structure, however slight these may be; and hence every disease has been essentially 'dynamic,' before it has become organic." All organic lesions he has reduced under the six following orders;—

1. Deviations from the normal nutrition of parts, either by excess (hypertrophe) or by defect (atrophy.)
2. Change of the position and connexion of parts.
3. Rarefaction of the tissues.
4. Generation of new tissues, which resemble some of the natural tissues, or homoioplasia.
5. Generation of tissues, which do not resemble any of the natural tissues, or heteroplasia.
6. Generation of morbid products, which may be either organised, and of independent vitality, or quite inorganic.

Our readers will find no difficulty in discovering the individual members of these six orders, except perhaps those of the third, which includes what M. Lobstein has designated "rarefaction of the tissues:" by this term he means "the diminution of cohesion between the component molecules of any part, by the interposition of a gaseous or liquid matter in the interstices of the solids." "It is," says he, "the most simple of all the alterations of texture, and by it all organic diseases commence: there are five species of it, viz. *pneumatosis,* the disengagement of a gaseous fluid; *hydranosis,* the transudation of a watery fluid; *hematonosis,* the transudation of blood; *fluxion,* or that vital movement by which the blood is impelled with unusual force, and in unusual quantities towards any point; and lastly, *inflammation.*"

In reference to this last-mentioned morbid process, which has of late years been invested with such an exclusive supremacy over every pathological change, the following very just observations are made by our author. "If inflammation was the source, and essential element of all organic lesions, these lesions, we may fairly presume, should generally occur in sanguineous temperaments and chiefly during the period of youth, when the organs of the circulation are in the most active state. Now the opposite of this is well known to be the case; for these very lesions depend in most cases on an irregularity of the nutritive process, which again is attributable to a 'vicieuse et languissante innervation.' How many of the most characteristic organic changes, such as aneurism of the heart, scirrhus of the stomach and of the womb, congestions of the abdominal viscera, and pulmonary consumption itself, are owing primarily to the baneful operation of some mental emotion! With the single exception of active aneurism of the heart, I do not remember to have met with one case of the fore-mentioned diseases in robust and plethoric subjects, but almost always in delicate feeble persons, whose health has been undermined by severe, or long-continued grief. Laennec has made a similar remark, relative to the disorganising influence of depressing mental affections: they derange all the functions, and in an especial degree, that of nutrition. It is a cheering reflection, drawn from these considerations, that not a few cases of well-marked organic disease have been cured by a treatment directed chiefly to the establishment of a healthy nutritive process in the system.

I have seen tumors gradually disappear, indurations dissipated, ulcers healed, in a word, cures almost marvellous, by the employment of mercurials, antimonials, sulphur, &c. and above all, of thermal waters. Formerly I was ignorant of the astonishing virtues of these waters, and did not believe that they exercised so penetrating an influence on the intimate structure and composition of the body. How often, when I have witnessed cases of disease deemed quite hopeless, gradually cured, have I asked myself, whether some imponderable principle, like that which resides in the nervous system, might not exist in these waters, and impart to them properties 'en quelque sort vitales.'" There is much good sense in some of the preceding observations. The volume is closed with the author's views on the mode of death in disease. "There is," says he, "only one 'maniere de mourir,' and that is, the paralysis of one or of more of the principal vital organs. When the brain is paralysed, death is said to proceed from apoplexy; when the respiratory system, from asphyxia; when the heart, from syncope; and when the solar plexus, from abepithymia.". — *Traité d'Anatomie Pathologique; Strasbourg;* 1829.

---

New Preparation of Quinine, the Hydro-Ferro-Cyanate.

Dr. Gouzet the principal physician of the Military Hospital, at Antwerp, has traced the effects of this new preparation in a good many cases.

The reports which he has made of its effects are highly flattering. In three cases of ague, (a quotidian, quartan, and tertian,) a cure was effected by a single dose, one grain of the salt, given an hour before the expected paroxysm. (If this is to be credited, the hydro-ferro-cyanate must be indeed a potent remedy.) Our author, who seems to be an ingenuous man, admits that the intermittent fevers prevalent at Antwerp, are often so mild in their character, that rest in bed, and "un regime très leger," continued for several days, are sufficient for their removal. The salt is prepared by boiling in distilled water a mixture of pure quinine and Prussian blue, and evaporating it to dryness.

The preceding extract is from the "L'Observateur Medical Belge," a new foreign contemporary, which is published at Brussels. It promises to be an exceedingly good periodical, and reflects much credit on the editor for the value of its contents, and on the publisher for the beauty of its typography.

---

VARICOSE TUMOR ON THE SCALP OF A NEW-BORN INFANT.—EXTIRPATION.—CURE.

The child was only six weeks old when Professor Mersseman was consulted: the tumor was situated exactly over the posterior fontanelle; it had attained the size of a goose's egg, was uneven and "bosselée" to the touch; the skin covering it was rather thinner than usual, but it was not discoloured, except when the child cried, or made any struggle, and then it became livid; there was no pulsation, nor alternate rising and depression of its surface; but when the hand was gently laid upon it, a vermicular sort of movement might be felt; its base was nearly as broad as the body of the swelling. This tumor had been observed at the period of birth, and was then of about the size of a pigeon's egg. The health of the child was otherwise perfectly sound and robust, and there were no symptoms of any cerebral disturbance. There was a difference of opinion among the medical men who saw this case, as to the nature of the tumor: by some it was supposed to be an encephalocele, or hernia cerebri; but the absence of all symptoms indicating any disorder of the nervous system, even when pressure was made upon the swelling, was unfavourable to this idea: that it was not caused by the protrusion of the dura mater, in consequence of a hydrocephalic effusion beneath, was, for the same reasons, as well as from other very obvious considerations, quite improbable: the absence of any pulsatory movements, and the fact that there was no increase of heat in the swelling, pronounced it to be not aneurismatic. Although the diagnosis was certainly obscure, Dr. M. was satisfied that the venous system entered largely into its formation, if it did not entirely constitute it; and he was led to this opinion by the varix-like unevenness of its surface, by the vermicular movements felt on gentle pressure, and by the circumstance of its distention when the child cried, or struggled, so that the return of the blood was somewhat impeded. Under this impression, he considered that the safest mode of treatment would be by a ligature put round the base of the tumor, and gradually tightened, until it sphacelated off. Fortunately, no unpleasant symptom followed the application of the ligature; it was tightened on the third day, and not till then, did the integuments present any appearance of strangulation: in other 24 hours suppuration had commenced all round the line of the ligature, and as the discharge was rather fœtid, pledgets wetted with the solution of the chloride of lime, were kept constantly on the part. By the fifteenth day, the pedicle being then not thicker than a swan's quill, a ligature "de sureté" was applied, and with one stroke of the knife the mass was detached: scarcely a drop of blood was lost. The exposed surface, (at the bottom of which the fontanelle might be touched) began speedily to granulate, the ligature "de sureté" dropped off, and in the course of a few days the sore was cicatrized.

On dissecting the tumor, it was found to consist of a bundle of veins, which had become enormously enlarged, so that at some points they formed almost spherical dilatations: these veins were joined together with an adipose cellular tissue, which had been converted, in consequence of the sphacelus, into a purulent pappy substance. The important lesson was afforded by the examination, how dangerous it would have been to have either incised or extirpated this swelling.—*L'Observateur Med. Belge.*

---

### Serum of a Blueish Colour from a Blistered Surface.

An anasarcous patient was recently received into the Hospital of Louvaine, in whom this curious phenomenon was observed.

After the use of diuretics (the particulars are not stated) a blister was applied on each ham, and the surface was then dressed twice a day with the "onguent perpetuel." The blue exudation was noticed first on the left leg, and in a few days subsequently, on the right one also : this coloured exudation continued for several days, after which, the discharge resumed its usual appearances.

Upon analysing the blue serum, it was found that its specific gravity was rather greater than that of water, that its smell was sickening and offensive, and that it had somewhat of a urinous taste. On exposing the linen stained with it to the action of the air and light the colour changed gradually from a sky-blue to a pale green. It was observed to affect test paper as an alkali does : this property depended on the presence of free ammonia. Acids converted the colour into a red. On evaporating the water, in which the linen had been steeped, a flocculent matter was deposited, and at length a residue of a deep green hue was obtained. The action of chlorine on the coloured water was very immediate, destroying at once all smell and colour; and these properties could not be restored by any means.

Several cases in which the urine or perspiration has been of a blue colour are recorded in different medical registers; and we are told that chemical analysis has always detected the presence of prussic acid, in union with some basis, under these circumstances.

In the one now detailed, no traces of any prussiate could be discovered.—*Ibid.*

---

### Anecdotes of the Distinguished Anatomist Vesalius.

With a praiseworthy spirit of national enthusiasm, our cotemporary of Brussels has commenced a series of biographical sketches of the most illustrious physicians and surgeons, whom Belgium, as their birth-place, lays claim to.

First and foremost on the list, is Vesalius, (born at Brussels, 1514,) the founder of human anatomy, and of whom Senac has said, "before he had completed his twenty-eighth year he had discovered a new world," meaning, the microcosm of man's body.

Before his time, the works of Galen, who it is well known, derived all his knowledge of anatomy from the dissection of the lower animals, were received as oracular; but their glory and ancient authority were doomed to cease, on the appearance of the splendid "Epitome de Corporis Humani Fabrica," published at Basle, 1542.

So admirable is the drawing of many of the figures, that it has been attributed to the pencil of Titian, who lived on terms of great intimacy with Vesalius, and whose beautiful portait of his friend was lately bequeathed by M. Portal to the Royal Academy of Medicine, at Paris. His countrymen were so justly proud of the magnificence of the work, that the magistrates of Antwerp, in 1572, voted a sum of the public money to complete the edition begun by the famous printer, Chrystophe Plantin, at his own expense. Vesalius commenced his studies at Louvain. He early distinguished himself by his excellence in the Greek and Latin classics. Repairing then to Montpellier, at that time celebrated as the chief depository of the Arabian literature, he pursued his medical studies with great ardour for some years; he afterwards went to Paris, as it afforded a more extensive and varied field for the prosecution of his favorite study, anatomy. There he became acquainted with Silvius, but a quarrel soon took place between them on the merits of oracular Galen, who was the "all in all" of the Parisian professor.

At this period, the war which had for so many years been carried on by Charles V. of Spain, against Francis I. of France, became more inveterate than ever, and the subjects of the one prince could not with safety remain in the dominion of the other. Vesalius therefore left Paris for Louvain, where he began to teach anatomy, and was soon after appointed surgeon of the Imperial army.

Although only 24 years of age, so high did his reputation stand, that he was solicited by the Venetian Government to accept the vacant chair of anatomy at Padua. He remained there until 1545, when he was summoned to Madrid, and made chief physician to Charles V.

The constant attendance which he was obliged to bestow on his illustrious patient, accompanying him, as he did, in all his enterprizes and campaigns, and the many ceremonials of a courtier's life, not to speak of the extreme difficulty of prosecuting dissection in a country so bigotted as Spain, were unfavourable to the accomplishment of those works, which Vesalius had engaged in, and which, imperfect though they are, remain proud monuments of his genius and perseverance. After Charles made the memorable abdication of his throne, in favor of Philip, his son, the same lofty distinctions were conferred by the young Emperor, on his father's physician; and if we may judge from the records of his great skill, these honors were fully merited. By his admirable knowledge of the structure of the human body, he had obtained a singular exactitude in the diagnosis of many diseases, the nature of which was but little understood in those days.

While in the plenitude of his fame and affluence, he was, as too generally happens, the object of rivalry and envy to others, and willingly was any report, however false and malicious, credited and diffused to injure the reputation of Vesalius.

Unfortunately an apparently just occasion was now afforded to these calumnies.

It happened that he had been attending a Spanish nobleman, whose disease had baffled the skill of all the medical men of Madrid. At the dissection of the patient, the surgeon and his assistants (Vesalius was not present) were horror struck, we are told, on finding that the heart still palpitated; and in the rashness of their ignorance, they loudly accused Vesalius of homicide, before the tribunal of the Inquisition. Sentence of death was pronounced by the monsters of this court against their innocent victim; and no doubt it would have been carried into execution, had not the intercession of the Emperor, and of many of the nobles, prevailed to transmute the punishment of death, to that of a pilgrimage to the Holy Land!! In 1564 he embarked for Jerusalem, and while residing there, he received an earnest solicitation from the Venetian Government to return to Padua, to fill the anatomical chair, which had become vacant by the death of Vesalius's favourite pupil, Fallopius. Alas! the master was soon to follow: the ship on the voyage home was shipwrecked off the coast of Zante, and he who had lately basked in the sunshine of the most splendid of the European courts, died of starvation on a desolate rock in the Mediterranean.

All the works of Vesalius were collected together and published under the care of Boerhaave and Albinus, in two folio volumes, at Leyden, 1725. It may be mentioned, that Copernicus, Galileo, Torricelli, and Pascal, were cotemporaries of the subject of the preceding sketch.—*Ibid.*

---

### Ligature of the Subclavian Artery. Patient survived 35 Days, although the Aorta was Diseased.

The patient was a man 44 years of age, whose health had been much injured by frequent syphilitic disease. There was a well-marked aneurismatic swelling of the subclavian artery, where this vessel is situated sacrad to the clavicle; and by pressing the finger into the hollow between the sternal ends of the sterno-mastoid muscles, the arch of the aorta might be felt beating violently.

The patient suffered great pain in the site of the swelling, and along the whole extent of the left arm; this was no doubt attributable to the compression of the axillary plexus of nerves; but there were other symptoms present of far more urgent importance, and which implied an unfavourable condition of the thoracic viscera: the patient had laboured for a considerable time under a distressing dyspnœa; the carotids beat most furiously, the features of the face were always full of anxiety, and sometimes severe headaches were added to his other sufferings. These circumstances induced Dr. Sentin to suspect the existence of disease either of the heart itself, or of its large vessels; and he therefore was opposed to the performance of an operation. The Valsalva treatment was tried for a week or so, and also permanent compression on the affected subclavian, where it passes over the first rib; but instead of doing good, these means seemed to aggravate the disease: the pain became now most excruciating, and the size of the aneurismatic swelling increased: the slightest movement of the arm caused the patient to scream out. The operation of tying the artery was now performed with the view, we are informed, "only of prolonging the poor man's life."

The symptoms of pectoral distress were, for two or three days after the operation, decidedly relieved, and the swelling was considerably reduced: a sharp darting pain was occasionally felt in the left side of the chest, but this was always readily removed by the application of the cupping-glasses. The wound suppurated, but the discharge of the matter was not free and copious.

On the 20th day after the operation the ligature came away, and every thing promised a complete recovery: thirteen days however after this date, a hæmorrhage to the amount of 8 or 10 ounces took place: this was arrested by moderate pressure, and as the pulse had become full and firm, two small venæsections and the rest of the antiphlogistic treatment were immediately practised: these means seemed to be successful, until the following day, when the hæmorrhage recurred: it was again arrested; but although the patient did not lose above ten ounces of blood, he became so exhausted, that all hopes of saving his life were dissipated. In the course of the ensuing night he died.

*Dissection.* A few ounces of serum were found in the pleuræ; the lungs were healthy; the heart was loaded with fat; traces of old inflammatory attacks were observable on the right ventricle; the calibre of the aorta from its origin till it reached the diaphragm, was greatly enlarged, and here and there the tunics presented patches of structural degeneration; the root of the left subclavian artery was distended into an aneurismatic sac, which adhered intimately to the apex of the superior lobe of the lung on that side; on tracing the artery, no coagulum was found within it, on the proximal side of the ligature, (which had worked its way through all the coats of the vessel) but at the distance of about two lines from the ligature was a perforation through all the tunics, which thus established a direct communication between the interior of the artery and the outer wound. It had been from this opening that the fatal hæmorrhage had escaped.

Considering the great extent of arterial disease, the temporary success obtained in the preceding case by tying the subclavian was certainly highly satisfactory.—*Ibid.*

---

Cases of Fracture of the Carpal Extremity of the Radius, with Practical Remarks on that Accident.

Case 1. *Fracture attended with Displacement radiad—Reduction on the 20th day.*

A lady, 69 years of age, fell with violence on the hand: the true nature of the accident was not recognised; it was believed to be only a sprain. As the swelling subsided the deformity of the wrist became more and more conspicuous, and the movements of the joint still more impeded. When she consulted Dupuytren at the end of the third week, the following was the state of the case;—the hand was strongly drawn outwards, or in the direction of abduction: a depression might be felt at the inferior extremity of the radius: the acts of pronation and supination were scarcely possible, and when attempted, caused extreme pain.

The replacement of the fracture was effected by an assistant grasping the forearm and making counter-extension, while M. D. drew the hand powerfully inwards, or in the line of adduction: by degrees the depression became less and less perceptible, and when the reduction was complete, the joint was well secured by the application of splints, which were kept on for ten days, and then re-applied for other three weeks. The cure was most satisfactory, no deformity remaining.

Case 2. *Fracture in a Child—Accident mistaken—Reduction* 29 *days afterwards.*

This patient received the injury by falling from a tree; the surgeon under whose care he was placed, thought it merely a severe bruise: the deformity became more apparent as the swelling declined. The fore-arm being secured by an assistant, M. Dupuytren was obliged to continue his exertions for a length of time before the depression between the fractured ends of the radius was effaced. At length however this was effected, and splints were applied: the application of these was renewed every ten days for three weeks. The cure was complete.

Case 3. *Circumstances similar to those in the preceding case.*

A boy, 13 years old, in consequence of falling from a tree upon his right hand, fractured the extremity of the radius; but this accident was not detected till the beginning of the fourth week after its occurrence. The reduction was quite as difficult as in the preceding case, and fortunately the event was satisfactory, the boy recovering with the perfect use of the hand.

Case 4. *Fracture—Nature of the accident mistaken—False joint formed.*

A middle-aged man came to the Hôtel Dieu to consult M. Dupuytren respecting an injury of his wrist, which he had been told, was dislocated backwards. The accident had happened three weeks before, and had been caused by falling on his hand. Several attempts had been made to reduce this supposed dislocation, but the deformity had always returned as soon as the extension was withdrawn.

Dupuytren at once recognised the nature of the injury. The radius had been broken immediately above its styloid process. The motions of the wrist and fingers were extremely painful, and indeed scarcely possible; but it was observed that the broken ends of the bone were freely moveable upon each other, and the Baron therefore inferred that a pulse, or secondary articulation had begun to be formed, no doubt in consequence of the repeated and violent efforts which had been made to reduce the deformity. He advised the patient to go into the Hospital as an in-patient, but this was declined, so that the event of this case is not known.

*Reflections on the preceding Cases.* It is the opinion of Dupuytren and some other excellent surgeons, that genuine simple dislocation of the wrist-joint very rarely, or perhaps never takes place, and that all the cases recorded as such have been so many examples of erroneous diagnosis. We do not at present include those cases, in which the joint, or its capsule have become diseased, as from white-swelling, dropsy, &c. Perhaps, under these circumstances, the extremity of the radius may become fairly displaced from the convex arch of the carpal bones; but such an accident must be very rare; all that we insist upon is, that it is seldom or never caused by outward violence. The injuries which have been mistaken for it, are dislocation of the ulna, dislocation of some of the carpal bones, fracture of the radius, or of the ulna, or of both bones, and lastly, separation of their epiphyses: by far the most frequent of these, is fracture of the carpal end of the radius. If these sentiments be correct, how countless must have been the instances of mala praxis. The Italian surgeon Paletta, arrived at the same conclusion, and nearly about the same period (1820) as the celebrated Frenchman; the words of the former are—"Suspicari liceat manum e loco penitus promoveri non posse." And again—"Ut diceres cubitum quasi ad id intentum esse, ne manus e suo nixu excidat."

The case recently published by M. Forget, in the Trans. Med. de Paris, as one of dislocation of the radius, we have no hesitation in pronouncing to have been rather one of dislocation of the ulna, and we have the authority of Sir A. Cooper to shew that such a mistake may not be uncommon: he says —"Luxation of the ulna at the first sight exhibits the symptoms of dislocation of the hand backwards." Certainly, in M. Forget's case, the symptoms reported do not warrant the conclusion which the author has come to, respecting the nature of the injury.

When in young children the extremity of the radius is broken, or what is more common, when the epiphysis is detached, the diagnosis is often still more obscure, than when the accident occurs in adults; the crepitation is much less distinct, and sometimes indeed scarcely perceptible, in consequence, no doubt, of the less density of the bone, of the greater quantity of blood effused between its extremities and of the periosteum, and indeed of many of the osseous fibres too, having not been lacerated; hence the lateral displacement of the fractured ends is usually less considerable, and hence too that degree of curvature which the wrist sometimes assumes in this accident, when it occurs in children. This last mentioned phenomenon is particularly worthy of attention, because, from not being well understood, it has given rise to a most serious practical error. It has been supposed that the carpal extremities of the bones of the fore-arm may, in young children become suddenly bent or curved without any fracture, or displacement of the epiphysis, by falling with force from a height on the hands: this accident has been denominated "primitive and accidental curvature of the bones of the fore-arm." The prognosis and treatment are thus described in a treatise, by M. Thierry, in a memoir published in 1808.

"Le mal guerit ordinairement en douze ou quinze jours, s'il n'est pas meconnu; mais, si l'on n'y apporte pas les soins convenables, l'avant bras reste difforme, s'atrophie, et le malade ne peut presque plus s'en servir."

"*Traitement.* Extensions tres fortes, mais graduées, et cooptation avec les paumes des mains pour redresser la courbure saillante."

We need not say that, however correct the prognosis and mode of treatment may be described, the diagnosis is quite at fault.

The following case, extracted from the late Professor Wilson's treatise on the Bones and Joints, is admirably illustrative of the truth of our remarks.

"A child, three years old, fell from a considerable height upon the pavement: the head was seriously injured, and the right fore-arm was bent nearly to a right angle. The child died soon after the accident; and on dissection it was found that the curvature of the fore-arm was the effect of a fracture of the bones: the fracture however did not extend through the entire thickness of the bones, the fibres on the posterior surface being not divided, and the periosteum moreover not being lacerated."

From the investigation of the two most celebrated surgeons of the present day, Sir A. Cooper and Baron Dupuytren, it appears, that there are three sorts of fracture of the carpal extremity; that the bone may be broken either right across, or in an oblique direction into the joint of the wrist, or into several pieces, constituting a comminuted fracture.

Now it is chiefly these two last-mentioned sorts, that have been so generally mistaken for dislocation of the wrist; and the error of diagnosis arises in this way; the wrist-joint, having lost the support and stability afforded by the articulating extremity of the radius, is drawn, by the action of the muscles of the fore-arm, from its normal direction, and becomes inclined either to one side or to the other, or forwards or backwards, dragging the fractured portion of the radius with it, and causing it to project, as if it were dislocated: the circumstances too of the absence of crepitation, and of the facility with which the deformity may be removed for the time by extension, tend to confirm the error of diagnosis. It is to be hoped that this practical error may be henceforth avoided by surgeons, as well for their own credit, as for the successful recovery of their patients; for it is to be remembered, that not a few awkwardly anchylosed joints have been the result of what have been supposed to have been cases of dislocated wrist. Sir A. Cooper judiciously recommends that the splints and bandages should be removed at the end of the third or fourth week, according to circumstances, for the purpose of accustoming the joint to gentle movements. Even under favourable circumstances the joint sometimes does not recover its free motion for many months: this inconvenience is most frequent, as we may suppose, after an oblique or comminuted fracture of the radius. — *Archives Generales, Aout,* 1834.

---

### PATHOLOGICAL ANATOMY OF THE PNEUMOGASTRIC NERVE.

Professor Albers, of Bonn, examined most attentively the pneumogastric nerves in forty-seven cases of hooping-cough: in four patients only, and these were scrofulous and lymphatic subjects, did he find any appearance of inflammation, or of any other disease.

In fifteen cases of phthisis pulmonalis these nerves were found on dissection to be unusually developed, and one was considerably larger than the other. Messrs. Swan and Descot have made the same remark. But it is in that disease which has been called intermittent croup (the crowing respiration of English authors?) in which the pathological anatomy of the nervi vagi has been most satisfactorily investigated.

The late ingenius researches of Dr. Hugh Ley, of London, have created much interest respecting this perplexing subject; and certainly some of the subjoined cases seem to corroborate the opinions of this able physician.

CASE 1. *Intermittent Croup—Tuberculous Enlargement of the Cervical and Bronchial Glands; adhesion of the Par Vagum to these Glands.*

A child, two years of age, labouring under the symptoms of croup, was visited by Dr. Hankel, in June, 1820. The glands of the neck were observed to be swollen. Three children of the same family had been cut off by sudden attacks of convulsion. Leeches, emetics, and calomel powders were ordered. On the following day the croupy noise in breathing had ceased; respiration still hurried; cough not severe; and towards evening the child seemed to have quite recovered from every troublesome symptom. Next morning the strangling dyspnœa returned, and after continuing for an hour or two, again ceased. Frictions on the neck with a volatile mercurial ointment. For twelve days these alternations of repose and distress were wretched: the child became gradually weaker, and died.

*Dissection.* The trachea was completely surrounded with swollen and diseased glands: three of these glands were much larger than the rest: one was situated over the left bronchus, to which it adhered by a dense cellular tissue; it was the size of a large walnut, and when divided, exhibited all the ordinary appearances of a pulmonary tubercle; at one point the tuberculous substance was beginning to be "ramollie;" the second was quite as large as the former one, and was situated exactly over the bifurcation of the trachea; the third was on the right side of the trachea, and adhered intimately to the par vagum of that side; the nerve did not however appear to be at all altered in texture; the lungs presented tubercular and melanotic deposition in different parts; the mucous coat of the trachea and bronchi appeared sound.

Dr. Hankel thinks it probable, that the attacks of croupy dyspnœa were owing to the irritation caused by the ramollissement of the glands, and that the death in this case "fut le resultat de la paralysie complete du nerf vague." It is probable, says Dr. H., that, much more frequently than we have hitherto supposed, the etiology of many thoracic diseases, and more especially those which are chronic, and which depend upon a morbid affection of the bronchial surface, is to be sought for in the condition of the cervical and bronchial glands. M. Andral also has directed the attention of pathologists to the same subject. The following case reported by him is beautifully pertinent.

Case 2. *Recurrent Dyspnœa—Death—Disease of a Par Vagum.*

A man, 24 years of age, had for a length of time suffered from most distressing dyspnœa: the respiration was always short, hurried, and confused, and to appearance was effected by the action of the thoracic muscles alone, the lungs being merely passive: the horizontal position was quite intolerable; the face was bloated; the lips and alæ nasi of a livid hue; the eyelids œdematous. No sign of any disease of the heart could be detected by the stethoscope. During a paroxysm of severe dyspnœa this patient suddenly expired.

On dissection, a portion of the left par vagum was found quite enveloped in a mass of tuberculous glands, and the substance of the nerve itself was extremely indurated.

Case 3. *Dyspnœa—Remarkable slowness of the Pulse—Death—Disease of the Nervi Vagi.*

Maria Cocchi, aged 40, had enjoyed good health till her thirtieth year, when she had a sevère attack of pneumonia: from that period the breathing had been always oppressed.

In October, 1832, having fatigued herself more than usual, and taken rather more wine than she had been accustomed to, she was seized with a stupor, which lasted for several days, and during which, she was scarcely conscious. On recovering from this attack, the respiration was more difficult than ever, and the pulse which was naturally slow, became extraordinarily so, beating only 24 times in the minute.

The chest being carefully examined with the stethoscope, the respiratory murmur was heard over every part; it was rather puerile: the sounds of the heart were regular, somewhat stronger, and might be heard over a greater extent than in health: the inference therefore was, that there was dilatation with hypertrophy of this organ. The diagnosis was certainly puzzling. A variety of remedies was tried, but with no good effect. Before death, the breathing became stertorous, and the face as purple as if she had been strangled.

*Dissection.* The head was examined, but no decidedly morbid appearances found there. In the chest there were several recent pleuritic adhesions: the principal bronchial glands were greatly enlarged and loaded with a calculous matter: a considerable quantity of a yellow coloured serum in the pleuræ, and also in the pericardium: the texture of the heart quite sound, but its volume somewhat enlarged: a few patches of steatomatous degeneration were seen on the aorta: the pneumogastric nerves, a little below the larynx, were found imbedded in a mass of swollen glands, and these enveloped in cellular membrane, which was much loaded with blood: the right nerve seemed to have been most affected, and at one point it presented the appearance of having been "tiraillé" and flattened.

CASE 4.—*Recurrent Dyspnœa—Vomiting—general Nervous Disturbance—Disease of the Spinal Cord and Nervi Vagi.*

A man 29 years of age, of an exceedingly nervous temperament, was, for a considerable length of time, distressed with fits of dyspnœa and of vomiting; these attacks were usually attended with a sense of constriction in the region of the stomach and across the chest, and with a numbness of the fingers and toes; subsequently, an annoying cough, pain in the throat, hoarseness, and paralytic weakness of the extremities came on, and, at length, the patient died semi-delirious.

On dissection, the spinal marrow was found softened, and otherwise diseased, at several points; the neurilema of the left par vagum presented a remarkable degree of redness; the cervical portion of the nerve was larger than usual, and seemed as if it had been soaked in a fluid, for its pulpy substance melted away between the fingers; these morbid appearances were especially distinct in its posterior or pulmonary branch—on cutting it across, several drops of serum escaped from the surfaces. The neurilema of the right nerve, also, was highly congested, but its substance was normal. The lungs, on the whole, were healthy; here and there, indeed, they presented a few hard tuberculous points; the heart and large vessels were sound—the abdominal viscera, with the exception of the stomach which had become so much softened towards the large end that it tore across on merely lifting it, exhibited nothing unusual.

CASE 5.—*Recurrent Dyspnœa—Dropsy—Death—Dissection.*

A man, 35 years of age, had, about 12 years previously to his admission into the hospital, suffered from a smart attack of hooping-cough, and subsequently from a spasmodic affection of the chest, which left him so irritable, that the least irregularity of the weather brought it back. A sense of constriction across the chest was seldom absent; the more severe attack usually set in by the respiration becoming short and oppressed—by a feeling, as if something was drawn tightly round the chest—by the voice being weak, and the effort to speak even painful; by degrees, these symptoms were greatly aggravated, and the dyspnœa was so grievous, that the surface of the body became cold and clammily damp, the voice shrunk into a hoarse whisper, the features were distorted, and the patient fell into a state of partial insensibility; the duration of these paroxysms varied from an hour to a whole day. In course of time, other miseries were added to this patient's sufferings; the face became bloated—the extremities œdematous—there was a sickening oppression at the epigastrium—the spleen was enlarged—an icteric yellowness of the skin, and, lastly, general dropsy supervened. In this state, the patient lingered for several years; every now and then, he seemed to have almost conquered his disease; but at length he fell a victim to its repeated attacks. For a few days before his death, there was a convulsive affection of the upper extremities, and a degree of general stupor.

*Dissection.* Substance of the encephalon healthy; serum effused between the dura mater and arachnoid coat; about an ounce in the lateral ventricles; lungs normal; several pounds of serum in the cavity of the peritoneum; abdominal viscera not materially affected in any way; the right par vagum, in its passage from the neck into the thorax, was highly congested with blood, and its substance was much harder than is usual; the nerve, on the left side, was smaller than its fellow, and was so much softened in texture, that it melted down between the fingers.—*Rust's Magazin, tomes* 39 *and* 41.

---

ANATOMY OF THE LYMPHATIC SYSTEM.

Our only object at present, in alluding to this obscure branch of anatomy, is to recommend to the notice of our readers, and especially to public teachers, a very admirable and original work by Professor Fohman, of Liege, entitled, "a Memoir on the Lymphatics of the Skin, Serous Membranes, and of the Nervous and Muscular systems," and published there last year in 4to, with ten plates. The author distinguished himself as an able anatomist, in 1821, by his ingenious treatise on the communications which exist between the venous and lymphatic systems, and again, in 1825, by his History of the Lymphatics in Fishes. In 1832, he published a memoir on the Lymphatics of the Placenta and of the Umbilical Cord. We have heard that the dexterity which he has acquired in injecting and demonstrating the lymphatic vessels is quite remarkable; and the value, therefore, of his plates is much enhanced by the assurance, that they were all drawn from injected preparations in his possession. Mascagni, his most distinguished predecessor, contented himself, it is well known, by tracing the uninjected lymphatics, by means of a magnifying-glass : it must be obvious that this method can scarcely be trusted to.

Prof. F. has ascertained, that the ultimate termination of the lymphatics is not in simple and open radicles, but in anastomotic plexuses, which become finer and more delicate as they approach the surface, whether of the skin, or of the mucous or serous membrane; there are not, therefore, open orifices at the extremities of these vessels ; and, if any orifices exist at all, they must be in their parietes or tunics.

---

### Melanosis of the Eyeball.

The following description of the appearances observed in two eyeballs, which had been extirpated for this disease, is taken from the "Abhandlung über die Melanose des Augapfels" of Dr. Pruscha. In one, all traces of the retina, corpus vitreum, lens, and iris were gone, and nothing but a brownish-black substance, penetrated with numerous tough fibres, was to be seen; the portion of the melanotic growth, which had protruded through the cornea, fell off, and, when put into water, readily dissolved, imparting a brownish-black colour to it. In the other eye, the only part affected with the genuine disease was the internal surface of the choroid coat, which was of a greyish-brown colour, firm, and was very vascular; the retina and corpus vitreum were converted into a semi-fluid, brownish substance; the lens was slightly opaque, the iris had a dirty-blue colour, and the cornea was normal, but pushed somewhat downwards.

---

### Phrenology, Human and Comparative.

We avail ourselves of Professor Bouillaud's review of Vimont's splendid work, "Traité de Phrenologie Humaine et Comparée, avec un magnifique Atlas, in folio, de 120 Planches, conténant plus de 600 Sujets," to extract a few of the most interesting details. The following propositions embody most of the general conclusions.

1. The figure of the cranium, in the different orders of vertebrated animals, is amazingly different; but each genus has its own unvarying type, and this cannot well be mistaken for the type of any other. The members of every genus exhibit numerous modifications of form, according to the characters of the animals.

2. The form of the cranium being known, it is quite easy to ascertain that of the encephalon, except, perhaps, at one or two points, such as over the frontal sinuses, &c. and also in some cases of organic disease.

3. In man, the anterior part of the cranium is more developed than in any other vertebrated animal.

4. The inner surface of the cranium exhibits in many of the genera, as in man, the quadrumena, ruminantia, pachydermata, solipeda, and carnivora, numerous pits and depressions, which correspond with elevations on the surface of the encephalon.

5. The inner surface of the cranium in the rodentia, and also in all birds, is, on the whole, even and smooth; one portion may be more depressed than another; but there are not the irregularities observed in the crania of the animals mentioned in the last paragraph.

6. In birds, the internal surface of the cranium is in more immediate apposition ("se trouve le plus en harmonie") with the encephalon than in other vertebrate animals. The rodentia are next, in this respect, to birds; and then the smaller carnivora.

7. In birds, there is a greater symmetry between the two sides of the encephalon than in other vertebrate animals; the right half corresponding very accurately to the left one.

The higher we ascend in the scale, the less perfect is this symmetry—in man, it is least conspicuous.

M. Vimont is at issue with several of his most distinguished cotemporaries, such as MM. Serres and Desmoulins, as to the existence of certain laws, or rather as to the truth of certain propositions, which they have enounced, relative to the physiology of the brain. It will be interesting to glance at some of these.

In the anatomie comparée du cerveau M. Serres states—" It is certain, that in proportion as the neck is lengthened in an animal, or set of animals, so are the brain and spinal cord more slender, " effilés." On this statement, M. Vimont remarks that there are numerous examples quite inconsistent with this assumed law; for example, the brain of the heron is much larger, and the spinal marrow less " effilée" than in the parrot; and again, the brain and marrow of the domestic goose are larger and less " effilées" than in the buzzard.

*The second proposition* advanced by M. Serres is expressed thus. " There is no steady relation between the volume of the brain and that of the spinal marrow, unless we confine our observations to one portion only of the former, viz. the corpora quadrigemina; between these and the spinal marrow there appears indeed to be a uniform ratio of development; so that the volume of one being known, we may " determiner rigoureusement" the volume of the other."

M. Vimont expresses his surprise at the bold assurance of M. Serres, in propounding such a manifest error. " The spinal marrow," says he, " of the cuckoo is very small, and the corp. quadrig. very large; in the buzzard, the marrow is smaller than in the domestic goose, but the corp. quad. are larger. Examples to the same effect may be derived from the anatomy of many of the mammalia."

*The third proposition* is, "that the development of the corp. quadrig. is in all the classes of vertebrate animals in proportion to the bulk of the eyeballs."

M. Vimont adduces several instances where this pretended law is quite at fault.

*The fourth proposition* is, " that the spinal marrow is in all the classes developed in a direct ratio to the size of the median lobe of the cerebellum." " Never was any dogma more erroneous," exclaims M. Vimont. Let us now turn to M. Desmoulins, and glance for a moment at some of his supposed discoveries. In the "Anatomie des Systemes Nerveux des Animaux Vertebrés," we are informed that " in almost all the species of the martin tribe, the brain has a smooth surface; there is not a single furrow on the brain of the weasel." M. Vimont replies, that surely M. D. has never dissected the brain of a single species of this genus; for in all, without one exception, the convolutions of the brain are very distinct, and he refers us to two of his published plates.

At another place of his work M. D. affirms, " that he has never been able to detect the least rudiment of a pineal gland in any of the carnivorous or of the gallinaceous tribe of birds." It is not surprising, says M. V. that a person who could not see any convolutions in the brain of a martin, has not been able to find the pineal gland in certain birds: it is distinct in the brain of the goose, the buzzard, the common fowl, and many others, which I have dissected. As to its presence in the eagle, hawk, &c., I have not satisfied myself on this point.

We have been so much amused ourselves with the truly French spirit of the following remarks that we are tempted to extract them for our readers' benefit. "I am certain," says M. Vimont, "that the brain of Napoleon must have been very large, because his head, independently of its own ample dimensions, belonged to a body in which the osseous system was not strongly developed, as we may infer from the well-known "petitesse de ses mains!" Professor Bouillaud is not inclined to be "seduit par cette raison." "I do not doubt," says he, "that Napoleon le Grand had a brain, 'qui fit honneur à son genie, ainsi qu'à la doctrine phrenologique,' but at the same time I am disposed to believe that the bones of the 'imperial cranium!' had an average thickness, for we find that the mould taken by Automarchi 'of the hero of St. Helena!' after death is remarkable for the 'beau developpement' of the lower jaw; and it is probable that the other bones of the face, and those of the cranium, corresponded in thickness with the lower maxilla!!"

---

### Detection of the Matter of Miasmata in the Air.

In a memoir lately read by M. Boussingault, at the Academy of Sciences, this most curious subject of meteorological enquiry is very ingeniously treated. It is well known that miasmata are almost always generated in places where dead vegetable matter is exposed to the conjoined influences of heat and of moisture, and hence, that they are of frequent and perhaps constant occurrence in warm marshy districts, especially if there be any admixture of fresh and salt waters, or if the ground has recently been turned up. The observations of all enquirers agree in representing the evening, soon after the setting of the sun, as the period at which the miasmatic influence is most actively pernicious, and in shewing that the deleterious atmosphere rests chiefly in the valleys and low grounds, while the more elevated positions are often quite exempt from its agency. Many years ago Moscate was led, by attending to these circumstances, to believe that perhaps the matter of miasmata might be discovered by collecting and condensing the dew, and submitting it to chemical analysis. His experiments were performed in the rice-grounds of Tuscany; and the results of these were, that the water so treated did very often exhibit flocculi, which had the properties of an animalised substance. Subsequently M. Delille announced that the water which he collected by condensation from the atmosphere in the marshes of Languedoc readily putrefied, and exhibited then flocculi of a highly azotised matter, which, on the addition of nitrate of silver, afforded a purple precipitate.

In 1819, M. Boussingault observed that sulphuric acid placed in the neighbourhood of pools, in which hemp was steeping, quickly assumed a dark colour, whereas, at a greater distance from these foci of putrefaction, the change took place much more slowly. At the period of this observation, a pestilential fever was ravaging the district of Ain, where M. B. was residing; and he was hence led to conjecture that the two coincident phenomena might be owing to the operation of the same agent, and that this agent, from its blackening sulphuric acid, no doubt, contained some carbonaceous matter.

When M. B. visited South America, he repeated these experiments in many places, which were notorious for their pestiferous miasms; but in these warm regions, the atmosphere so teems with insects, that the change in the colour of the acid might be supposed to be owing, in part at least, to some of these having fallen into, and being charred by, it. His apparatus, therefore, required some improvement, to guard against this objection, and he acquaints us that when he was residing at Carthagena, he adopted the following precautions:—

"Soon after sunset," says he, "I placed two watch-glasses on a table in the middle of a marshy plain: in one of these glasses I put a few drops of warm water, for the purpose of moistening its surface, and imparting to it a temperature somewhat above that of the atmosphere: the cold glass was speedily covered with dew, but almost none was condensed on the other one. A drop of strong sulphuric acid was then added to both glasses, and when their contents were slowly evaporated to dryness by the heat of a lamp, it was invariably found that a trace of carbonaceous matter adhered to that glass in which the dew had been deposited, while none could be seen in the other. This mode of experimenting possessed the advantage of requiring but little time for its performance, and if any insect happened to fall into either glass, it could be at once removed with a needle.

By using two glasses, the objection that any particles of dust floating in the atmosphere might have been entangled in the fluids was guarded against; for if the carbonization depended upon this cause, it is evident that it must have affected both glasses equally."

The above-mentioned experiment was repeated by M. B. for several evenings, and uniformly the same results were obtained.

---

RUPTURE OF VARICES OF THE VAGINA AND EXTERNAL GENITALS, DURING AND AFTER LABOUR.

This accident, although of rare occurrence, is truly alarming, if it takes place at the period of parturition. The narration of a few cases will best illustrate its character.

CASE 1. *Rupture of the Varicose Tumor—Death.*

A woman, 41 years of age, pregnant with her fifth child, was seized with labour-pains on the evening of the 17th August. When the membranes had burst, and the liquor amnii was discharged, the pains gradually abated. Soon afterwards, however, upon the patient being placed " sur le lit de travail," a severe and lengthened pain came on, and the midwife now found that the left labium had become immensely swollen and discoloured. The physician was immediately summoned; but, before his arrival, the tumor had burst, and a quantity of black blood been discharged. The woman became immediately exhausted, then convulsed, and she died within half an hour after the accident. The child was extracted after the death of the mother, but it also was already lifeless.

*Dissection.* On the inner surface of the left labium was found a rent, an inch and a half in length, through which the fingers might be passed into a " vaste poche," containing a considerable quantity of clotted blood. On the right labium, and on the inner side of both thighs, were several varicose swellings, which no doubt would have burst at some future period, had the patient lived, and been exposed to strong efforts of any kind.

CASE 2. *Rupture of the Varix—Death.*

The labour had continued for upwards of twenty hours, when the " sage femme" found to her consternation that the right labium was much distended and discoloured, and that blood was dripping from its inner surface. Notwithstanding her alarm, she did nothing more than merely apply a wet rag to the " bas ventre." In a quarter of an hour this poor patient " avait cessé de vivre." The doctor, on examining the parts, found a rent, two inches long, on the inner surface of the right labium; it led into a sac, of nearly four inches in circumference, and which extended under the os pubis, and on the outer side of the ascending ramus of the arch: at the bottom of this sac was seen the ruptured orifice of the varix. This patient was about 40 years of age. The pelvis was somewhat contracted in its dimensions.

CASE 3. *Rupture of the Varix—Death.*

Dr. Ebert was summoned to the assistance of a woman in labour, but she had just expired on his reaching the house. She was about 34 years of age, and the mother of seven children. The labour had begun favourably, and the pains had been at first regular and steady. On a sudden the woman felt that a quantity of fluid was running away from her, but she thought that it was the " waters;" the nurse, however, found that it consisted chiefly of blood: in half an hour the patient "rendit le dernier soupir." The corpse presented an exsanguine appearance, the face and extremities being blanched like white wax. On the right labium, which was considerably swollen and of a livid colour, were seen three ragged wounds; through each of these, the finger could be passed into a "cavité vaste," which still contained a quantity of black coagulated blood. On the left labium, and also on both thighs, there were numerous varicose distentions.

In the three preceding cases, the accident took place before the expulsion of the child, and in all, the child was dead when extracted. In the following case, the delivery had fortunately taken place, before the rupture of the varicose swelling.

CASE 4. *Rupture of the Varyx—Recovery.*

Immediately after the expulsion of the fœtus, the external parts became enormously swollen, and at length the integuments gave way at one point, and an alarming hæmorrhage took place. The immediate application of rags dipped in cold water arrested the flow of blood; and the exhausted strength of the patient was then supported and revived by sinapisms to both legs, and by a cordial medicine exhibited frequently.

CASE 5. *Varicose Tumor high up in the Vagina—Rupture—Death.*

A woman, 46 years of age, mother of eight children, suddenly expired in labour. Dr. Carus found on his arrival that the head of the child was detained in the outlet of the pelvis; he delivered it with the forceps, but not without experiencing considerable difficulty; for the dimensions of the pelvis were rather confined. On introducing the hand afterwards to extract the placenta, he found near the cervix of the uterus "une tumeur variqueuse rompue:" the rent was about an inch long, and the extent of the cavity about two inches all round. The head of the fœtus when extracted, was found covered with clotted blood.

*Reflections.* The preceding cases point out the characters of this species of puerperal hæmorrhage, which depends upon a varicose state of the veins of the vagina and neighbouring parts.—The distention of these organs during parturition and the consequent impediment to the free return of the venous blood, sufficiently account for the almost invariable occurrence of the accident either during its progress or immediately after it is over. Perhaps in most cases there is a simultaneous abridgment of the dimensions of the pelvis; or, at least, some existent cause of obstruction to an easy labour, either on the part of the mother, or of the child. We observe from one or two of the cases now recorded that the hæmorrhage may be speedily fatal; and indeed we cannot wonder at this when we consider that there is a free communication between the veins of the womb on the one hand, and of the thigh on the other, with those of the vagina.—*Med. Correspondenz-Blatt.*

---

### DR. BELLINGERI ON FACIAL NEURALGIA.

This distinguished physician of Italy informs us that, in his practice at the hospital of Turin, during the last 14 years, he has had 40 cases of facial neuralgia, affecting either the portio dura, or some of the branches of the trigeminus nerve. The relative frequency of the disease in these two nerves is, according to his experience, certainly very different; for we are told that there were only two instances of the former, viz. the portio dura, being involved; (in one of these it had been caused by a wound of the cheek, and in the other it was evidently connected with some enlarged scrofulous glands in the neighbourhood;) whereas in all the rest, 38 in number, the neuralgia was seated in the latter. The branches of the trigeminus most commonly attacked were the frontal, and the sub-orbital; and it is curious that, with regard to the former, the disease was almost always on the left side; and with regard to the latter, on the right side. When neuralgia affects the frontal or other branches of the ophthalmic nerve, it has, Dr. B. says, much more frequently a periodic or intermittent character, than when the suborbital nerve is affected.

Age appears to have a very considerable influence in predisposing to this disease; for, with the exception of two cases, all the others occurred in patients who had passed their fortieth year. The season of the year, too, has not a trifling agency on the phenomena of the disease. It is in Spring and Autumn that the disease is usually developed. As to the etiology of the cases which occurred in Dr. Bellingeri's practice, we are told that two were of traumatic origin—(in one it followed a fall on the head; in the other, a piece of iron had struck the cheek over the infra orbital foramen;)—that two were caused by sudden frights; (in both the disease was very obstinately prolonged, but at length spontaneously subsided in severity;) two, by the cessation of accustomed sanguineous discharges,—(both cured by an antiphlogistic and soothing treatment;) and that all the other cases were of a rheumatic origin, and attributable to the operation of cold and damp. Very frequently the first attack could be clearly traced to a residence in a low marshy district, or in a house recently finished, and whose walls were not thoroughly dry. Two priests, who successively inhabited the same house, (which was exposed to the westerly winds,) were both attacked with neuralgia, shortly after their residence in it, although they had never experienced the disease before.

A senator went out on the first day of January, 1814, in his carriage, to take a ride. The carriage window on the right side was left open; in the course of the evening he began to feel darting pains in his right cheek, and the disease, which speedily assumed a very severe form, has now continued for the last 20 years. For the first six years the paroxysms were very dreadful; of late, they have subsided considerably in intensity. Another similar case occurred in a lady, who had incautiously exposed herself at a window to a current of cold air. The attack of the disease was almost immediate. For the course of twelve years this patient was subjected to frequent returns of the neuralgia; at length it subsided spontaneously, and has not returned for the last three years.

Dr. B. recognises three distinct sorts or genera of neuralgia—the inflammatory or congestive, the irritative, and the purely nervous. The first of these sorts includes, as a general rule, all those cases that follow the suppression of the menstrual or hæmorrhoidal discharges, or are induced by exposure to cold, repulsion of exanthemata, by wounds of the nerve itself, and in which there is in all probability either a decided phlogosis or a sanguineous engorgement of the affected nerve. It is to be remembered, however, that the disease may continue, and in its most inveterate forms, after all inflammatory or congestive action has ceased. Under such circumstances, the neurilema or substance of the nerve itself has not unfrequently been found organically affected, either by thickening of their substance, or by serous infiltration into it. The following remark by Bellingeri deserves to be impressed on the mind of the practitioner. It has often occurred to him to cure neuralgia by antiphlogistic remedies, although the case had resisted the very same treatment at an earlier period of its course. The very reverse, however, of this proposition is generally found to hold true, for the more ancient and obstinate the disease has been, the less tractable is it usually by the medication now alluded to.

The irritative form of neuralgia, is that which depends upon the irritation of a nerve, either by the contact of some abnormal or foreign substance, such as a decayed tooth, an enlarged gland, &c.; or again, as our author says, "par la retrocession d'un exantheme, ou de dartres, ou enfin de la syphilis;" and he adds, "dans tous ces cas la neuralgie n'est curable qu'autant qu'on fait disparaitre la cause irritante, qu'on rappelle les exanthemes, &c."

The purely nervous neuralgia, (under which denomination we include all such cases as do not belong to the two former sorts,) has always a type more or less periodic, and therefore may be either regularly or irregularly intermittent. It is certainly not frequent that neuralgia assumes this form from the very earliest commencement of its attack, except in those cases where it suddenly supervenes upon a violent mental emotion; and, moreover, we are to remember, that although purely nervous at first, the disease may, and often does, become of an inflammatory or congestive character at some period of its progress.

As to the "treatment" of neuralgia, the experience of Bellingeri coincides in almost every respect with that of the best practitioners in this country. When the disease assumes a regular periodic form, it is very generally curable by cinchona, especially if, for a few days before the exhibition of this drug, antiphlogistic means and other necessary preliminaries be attended to. By far the greater number of cases in our author's practice were of an inflammatory character, and they almost invariably yielded to bleeding, &c. &c. Extract of henbane, in large doses, has been found by Dr. B. a most useful adjunct. In several cases a most decided benefit was derived from the endermic use of acetate of morphia, by sprinkling it upon a blistered surface. Half a grain every twenty-four hours is a sufficient dose at first; it may be gradually increased to two grains. (En passant, it may be stated that Dr. B. has often cured most obstinate hiccup in this way.) The following case is worthy of record.

A lady, 30 years of age, of a plethoric temperament, but of a very delicate constitution, after a residence of a few months in a damp house, began to experience severe neuralgic pains in the quadratus lumborum muscle of the right side. The pain extended down the corresponding limb, which at times was quite benumbed. The most intense agony was felt over the hip. These paroxysms of pain returned almost every day, and lasted generally for five or six hours, and then gradually subsided, but never ceased entirely;—there was always a feeling of distress in the parts;—no perceptible alteration was observable in the integuments. The treatment adopted at first consisted of rigorous antiphlogistic measures; the patient was bled five times, and the blood was found to be very strongly buffy; large doses of henbane, aconite, quinine, &c. were then exhibited, but all to no effect. At length, a perfect cure was effected by applying a large blister on the part most pained, and sprinkling the denuded surface with two grains of acetate of morphia. Syncope, nausea, and a most disagreeable pruritus of the forehead and nose were induced; but fortunately the neuralgia ceased, and it never returned afterwards.

When we have reason to apprehend any change of structure, either of the nerve itself or of its neurilema, such as thickening, serous infiltration, &c. frictions with the mercurial, the iodine, or the antimonial ointment, may possibly be used with advantage; and the internal exhibition of the kermes mineral, or of the red sulphuret of mercury in union with the extract of henbane or of aconite, should be conjoined. If these means fail, the section or cauterization of the affected nerve may be tried with some hopes of relief.—*Annali Universali di Medicina.*

---

### Professor Albers on Inflammation of the Spinal Dura Mater.

The pathognomonic symptoms of this affection are, according to our author's experience, acute pain, stretching to, and felt chiefly in the lower part of the trunk and inferior extremities; convulsive movements of different parts, accompanied with a trembling or tremulous agitation; difficulty in expelling the urinary and alvine discharges; and lastly, a feeling of tightness or constriction round some part of the body. We shall briefly allude to each of these sets of symptoms in detail.

1. The acute pain in the parts situated below the seat of the inflammation was observed in the examples reported by M. Funk, as well as in those observed by Professor Albers. This symptom has been repeatedly noticed in cases of tetanus. The pain is usually accompanied with a sense of pricking and tearing; and these distressing feelings are in most instances referred to the epigastrium, hips, and thighs. In very severe cases so acutely sensitive may be certain parts that even the slightest touch causes excruciating pain and cramps. Now this symptom, or rather chain of symptoms, is not observed, when the arachnoid and pia mater, or when the substance of the medulla itself is affected: under these circumstances, after the pain and convulsive movements have continued for some time, a paralytic weakness usually supervenes. On the contrary, paralysis is certainly not a common result or effect of inflammation of the dura mater, unless the disease has involved the parts situated within its sheath.

2. *Convulsive movements.* When the cervical portion of the spinal dura mater is affected, the muscles of the face and neck are usually drawn into irregular contractions, along with those of the chest and abdomen. The upper extremities are affected more tardily, and sometimes not at all, until a few days before death. It is not, however, unfrequent that severe and irregularly returning pains are felt from the shoulders down the arms; certainly the upper extremities are more generally affected than the lower, when the disease is situated in the cervical region. When the whole extent of the cervical section of the medulla is inflamed, the upper extremities are almost certainly affected; but when the inflammation is limited to its inferior portion, they may escape altogether, or exhibit only a trembling movement or agitation, while the lower limbs are all the time contracted with tetanic spasms. Sometimes the arms are spasmodically affected, while the thighs and legs are paralysed. Under these circumstances, we may expect to find on dissection an effusion of reddish serum within the sheath of the dura mater, at its lower part. As long as the quantity of this serum is small, it may act as an irritant on the cauda equina, and may thus cause convulsions of the inferior extremities; but when it becomes more abundant, it induces, in consequence of the compression, partial or total paralysis.

Professor A. remarks, that the tetanic convulsions which attend inflammation of the dura mater are almost always of the "tonic," and very rarely of the "clonic" kind. The cause of the persistance and intensity of the spasm is no doubt attributable to the irritation, which the diseased envelope exercises on the contained medulla, being constant and not remitting. "Now, this is not the case when the medulla itself or its immediate coverings are affected. The state of high excitement lasts but for a short time; hence the violent convulsions are either from the exhaustion of the nervous energy, or from the compression on the nervous mass, speedily followed by paralytic weakness. On the other hand, this state of paralysis seldom occurs in inflammation of the dura mater, except for a few hours before death."

3. *Tremulous agitation of different parts of the Body.* This symptom is usually most conspicuous in the early stages of the disease when the patient attempts to walk. He finds that he cannot keep his head steady for a moment, and that his arms, body, and legs are in a constant tremor. This is one sort of the "paralysis tremens" of Dr. Cooke. It may be observed, that the involuntary action of the muscles we are now alluding to, always ceases when the patient is in the horizontal position. As the disease lasts, this symptom becomes less and less distinctly marked, and either disappears altogether, when a favorable termination may be anticipated, or it is succeeded by a tetanic stiffness or contraction of the limbs. As soon as tetanus begins to appear, the tremulous agitation ceases.

4. *Difficulty in expelling the Urinary and Alvine Evacuations.* In general, the paralytic affection of the bladder precedes that of the rectum; and, indeed, the latter viscus is sometimes but little affected till within a short time before death. The true nature of the urinary local distress is not unfrequently mistaken at first, and it is only when the bladder has become enormously distended, that the practitioner is made aware of the existing evil.

5. *The Feeling of Constriction round the Body.* When the cervical portion of the medulla is inflamed, the sense of constriction is usually experienced round the lower part of the thorax, extending from the dorsal vertebræ to the epigastric region. When the lower part of the dorsal, or when the lumbar division is the seat of the disease, this symptom is remarkable chiefly in a line, running almost parallel to the anterior and superior crista of the os ilii. The sense of constriction is not unfrequently accompanied with positive pain; it is always an unfavourable sign, as it indicates the intensity of the inflammatory action. When once established, it is, even under the most fortunate circumstances, very obstinate of removal.

In concluding these remarks on inflammation of the spinal dura mater, we may state that the functions and faculties of the brain may remain uninjured, under the most serious disease of the medulla spinalis.—*Graefe und Walther's Journal fur Chirurgie, &c.*

---

Singular Case of Epileptic Hysteria, in which there was a strange Propensity to a rapid Progression, and then to Rolling of the Body during the Paroxysms.

A man, 26 years of age, of a sanguineous and extremely irritable temperament, and who had been much addicted to debauchery and other excesses, was ten years ago seized with convulsions of a very extraordinary kind. The paroxysms came on sometimes during waking—at other times during sleep; the patient would, all on a sudden, scream out, and lose all consciousness; he then began to run rapidly forwards, turning neither to the right nor to the left, nor stopping at obstacles which he could possibly overcome. This "course rapide," if not checked, continued for a few seconds; the distance which he usually went being 20 or 30 paces. On recovering from the attack, his mind seemed composed, and he was not at all aware of what had taken place; he only recollected that, when his consciousness was leaving him, he experienced a sort of "aura" creeping up from his toes, along the back, until it reached the head. During a period of nearly seven years, these curious paroxysms returned at irregular intervals; often, indeed, there were two in the course of 24 hours—at other times there was a much longer space between them. Their character became gradually altered, and, instead of the patient being impelled to a rapid progression, he now, during the attack, fell insensible at once on the ground, and began rolling himself fairly round about, for twelve or fourteen times; when this singular propensity ceased, his consciousness returned by degrees. During the continuance of the paroxysm, the patient kept constantly screaming out. For the last two years or so, these paroxysms have been more frequent, and also more lengthened; and usually, when the rolling has ceased, irregular and very bizarre contractions of different parts supervene. The attacks are more common at night than during the day, and while the patient is asleep than when he is awake. It is seldom that an entire day passes over without one or more paroxysms.—*Il Filiatre Sebezio.*

---

Neuralgia of the Leg and Foot—Section of the Sciatic Nerve.

A man aged 31, had his right foot injured by a wheel passing over it. Under proper treatment he quickly recovered from the effects of the severe contusion, and he experienced but little inconvenience for the space of five years. After the lapse of this time, on accidentally hitting the toes against a stone with considerable force, he was forthwith seized with a sharp, darting pain in the foot and leg ; this pain lasted only for a few minutes, and then it subsided; but, unfortunately, any movement of the limb threatened to re-induce it. From this date may be reckoned the commencement of all his subsequent sufferings. During the following three years, whenever he attempted to walk, he experienced a dreadfully lancinating pain on the dorsum of the right foot, and affecting all the toes: in the fourth year, the pain became still more severe, and extended to the sole of the foot and os calcis. The paroxysms usually recurred several times in the course of the twenty-four hours, and during their continuance the agony was almost insufferable. All sorts of treatment had been tried, but, alas! with very little relief.

In 1827, Dr. Malagodi first saw this patient :—he found the right leg considerably atrophied, but exhibiting no outward traces of inflammation, or other disease ; the attacks of pain usually came on without any warning. All on a sudden, the patient was seized with an excruciating pain, which forced him to stand still, and remain in the same attitude for upwards of an entire hour ; if he attempted to move, the limb became affected with spasms and convulsions ; he was obliged to use a crutch on all occasions. His general health had suffered materially—his appetite failed, and his sleep was much disturbed.

The circumstance of the utter failure of every remedy which had been tried, determined Dr. M. to perform the section of the sciatic nerve. An incision, about three inches long, was made through the integuments, on the lower and back part of the thigh ; the fascia lata was then slit up in the same direction, and the two masses of the flexor muscles being separated from each other, the nerve was exposed, and then divided in two places, viz. at the upper and at the lower angle of the wound. A tremor suddenly seized the limb, and the patient became faint; he afterwards described that he had felt a pain shoot from the wound along the vertebral column, to the head, at the moment of the division of the nerve, and that, immediately afterwards, he lost all consciousness. When he recovered his sensibility, he found that he had lost all power over the limb, and that there was a feeling of weight and of formication all over it. For eight hours after the operation, there was an entire absence of suffering ; then he felt a pricking twice or thrice in the foot, extending from the sole to the dorsum of the foot. On the following day, as he experienced a very severe pain on the outer side of the affected leg, a venæsection was practised. On the third day a feverish irritation came on, and this state lasted for five days: " on pratiqua six saignées," and exhibited purgatives and antimonials. During all this time, the pain in the right leg continued obstinately—sometimes it was felt in the wound itself, and along the course of the sciatic nerve. The repeated application of leeches to the limb brought great relief, and a decided amendment seemed now to have taken place. By the end of the seventh week, the patient was able to walk about, without experiencing any suffering, and, moreover, without the assistance of any support; his general health gradually became quite re-established. The wound was not entirely healed till the end of the fifth month after the performance of the operation.—*Annali Universali, Aprile*, 1834.

---

### TWO CASES OF SUCCESSFUL TRANSFUSION OF BLOOD IN UTERINE HÆMORRHAGE.

*Case* 1.—A woman, 41 yeas of age, and mother of several children, was seized after a suppression of the catamenia during three months, with a " perte uterine," which soon became alarming, from the profuseness of the discharge. It had continued for 18 hours, when Dr. Klett was called in. He found the patient in a state of extreme exhaustion; a vast quantity of blood must have been lost, as it had soaked through the bed, and quite inundated the floor.

Various cordials and stimulants were immediately exhibited, and astringent injections were thrown up the vagina; but the deadly paleness of the face and coldness of the limbs, the singultus and hiccuping, &c. sufficiently indicated the necessity of some other expedients being tried. Two ounces of blood (procured from the husband's arm) were immediately injected into the cephalic vein; the result was very surprising—the patient at once opened her eyes, the pulse became distinct and moderately strong, the syncope and hiccups ceased, and a pleasant warmth was diffused over the whole body. The medicines formerly used with no benefit, were now administered with the happiest effects, and it only required a steady perseverance in them to complete the cure. The patient, on being asked what sort of sensations she had experienced from the injection, replied—"qu'elle avait ressenti manifestement le mouvement d'une circulation de chaleur se portant vers le cœur, et qu'elle devait consacrer sa nouvelle vie à conserver la mienne; ce sont là ses propres expressions!!"

*Case* 2.—When Dr. K. was summoned to the relief of this woman, the flooding had lasted for upwards of ten hours. She was in a most alarming condition, and "faisait ses derniers adieus d'une voix foible et entrecoupée, et elle resemblait la morte."

About two ounces and a half of blood, drawn from the arm of the husband, who was "fort et bien portant," being injected into the median cephalic vein, "la vie parut se ranimer, comme par une commotion electrique." The recovery was slow, but ultimately quite complete.

---

### Professor Bouillaud's Refutation of Majendie's Theory of the Sounds of the Heart.

In some of our recent Numbers, we have alluded to the substance of M. Majendie's memoirs on the double sound, or tic-tac, which accompanies the alternate motions of the heart. The theories of Laennec himself, and, subsequently, of Drs. Bouillaud, Bertin, Hope, Corrigan, and others, referring the cause of this acoustic phenomenon either to the tremor of the muscular fibres of the heart, to the alternate closing and opening of some of its valves, to the impulsion of the current of the blood on the inner walls of the cavities, &c. have appeared unsatisfactory to the distinguished physiologist of Paris, and he has been, therefore, induced to push his own boat off into the waters of speculation, with the full hope, no doubt, of being a more fortunate fisherman of the truth.

M. M. is of opinion, that the tic-tac sound is owing to a "double choc qu'exerce le cœur sur les parois du thorax; l'un de ces chocs ayant lieu par la point de l'organe à l'instant de la contraction des ventricules, l'autre par sa face anterieure au moment de leur dilatation." This explanation has the merit of apparent simplicity, and one might, on a passing examination of the subject, be inclined to admit its accuracy; it becomes, therefore, and for this very reason the more necessary, to disabuse the minds of those who have not attended minutely to the physiology of the heart's action (a subject which, since Laennec's immortal discovery, has, perhaps, been too much insisted upon by some physicians, and occupied a too exclusive share of their attention) of the fallacies of M. Majendie's theory.

Professor Bouillaud has done this very ably, and we cannot do better than give a short abstract of his mémoir, in reply to M. Majendie's, recently read at the Royal Academy of Medicine.

We may premise, that the experiments of Dr. Hope, in the first instance, and subsequently of the Parisian Professor, afforded, among other results, the almost unanswerable objection, that the sounds of the heart were distinctly heard after the chest of an animal had been laid fairly open, and when, therefore, there could not possibly be any shock or impulsion against its parietes. In following out his argument, M. B. rests considerable weight on the occurrence of the unnatural or abnormal sounds, either merely accompanying, or altogether superseding, the ordinary tic-tac heard in a healthy state of the viscus; "for since (says he) the abnormal sounds are clearly referable to certain abnormal states of parts of the heart itself, and since the abnormal sounds often quite supersede, and, as it were, take the place of, the healthy or normal sounds, we may reasonably believe that the normal sounds, also, are due to the same cause." The causes of the abnormal sounds are threefold :—

1. They may depend upon certain organic changes of the lining membrane, or of the valves of the cardiac cavities : of these changes, the most frequent and best known is induration of the valves, and the consequent impediment to their free play, and the contraction of the valvular orifices. To this source we attribute the blowing, bellows, saw, rasp, file, and whistling bruits of the heart. M. B. says that the last-named bruit is sometimes so remarkable, as to resemble "le cri d'un oiseau."

2. Lesions of the pericardium may give rise to abnormal cardiac sounds; for example, when the two opposed surfaces of this envelope become uneven and irregular, the friction of the one surface upon the other may occasion either a sort of diffused and superficial rasping bruit, or else a sound resembling the crackling of new leather. When there is a considerable effusion into the bag of the pericardium, the cardiac sounds are rendered more dull, and they are heard as if they were more remote from the ear. In a case which was recently under M. B.'s care, a most distinct grating, or scraping sound was heard, during the whole time the patient was in the hospital; the patient died, and, on dissection, an osseous concretion, projecting from the surface of the pericardium, was found.

3. Lesions of the muscular substance of the heart, or of "son principe excitateur" (in consequence of which the impulsions of the organ against the thoracic parietes are rendered either more powerful or more feeble) modify considerably the ordinary cardiac sounds. As the impulsion is coincident only with the systole of the heart, this third set of abnormal sounds must, it is evident, correspond with the contraction, and not with the dilatation of the organ. In several cases, the sound, caused by the shock of the heart against the thoracic parietes, is of a remarkably clear, silvery, metallic resonance. In concluding his remarks, the learned Professor very justly re-urges his former proposition, "that if the sawing, rasping, and other such abnormal sounds, are modifications only of the healthy tic-tac of the heart, is it not reasonable to believe that this tic-tac depends itself upon the same essential or original causes, viz. the play of the cardiac valves, and the current of the blood through the valvular orifices."

M. Majendie, indeed, says that a narrowing of these orifices prevents sometimes the first—at other times the second, impulse or shock of the heart; and that it is to this cause, viz. the absence of the twofold impulsion, that we must attribute the absence of the first bruit, in the one case, and of the second bruit in the other. In reply to this statement, M. Bouillaud asserts (and his assertion is doubtless conformable with the experience of other physicians) that in a multitude of cases, in which the cardiac orifices were proved by dissection to be contracted, the impulses of the heart had been, during life, not only not impeded, but, on the contrary, had been much stronger than in a state of health.

Whoever will attentively examine for himself the acoustic phenomena of the heart's action, will be at once satisfied, that the sensations produced by the shock of the heart against the thoracic parietes are, although simultaneous, or nearly so, with the proper cardiac bruits, quite distinct and independent from them.

It is altogether curious to observe the conflicting discrepancy among medical men, not only in their trains of thought and reasoning, but also in their observation and perception of phenomena cognizable by the senses. One argument on which Majendie rests considerable weight, for support of his doctrine, is, that according to his observations, the "shock, as well as the sounds of the heart, when this viscus is hypertrophied, are always enfeebled, and sometimes altogether nullified," and the reason he assigns is, "qu'à aucun moment il n'existe plus de distance entre lui et la paroi pectorale." It is almost unnecessary to state, that the observations of other physicians are diametrically opposed to those of M.M.—*Journ. Hebdomad.*

---

### Secale Cornutum, as an Emmenagogue.

*Case* 1. A girl, 16 years of age, had for several months suffered from general malaise and cachexy; the abdomen had become tumid and hard, the limbs much emaciated; there was a complete anorexia, and great debility.

Every evening between five and six o'clock she had a pyrexial attack, which lasted for a few hours and then abated. This girl had menstruated only once in her life time, and that took place about two months before she was visited by Dr. Rollet.

As there was some degree of tenderness in the epigastric region; a dozen of leeches were applied there, and febrifuge medicines were administered, with the view of obviating the evening paroxysms; but these seemed after a few days perseverance in this mode of treatment rather aggravated than relieved: leeches were then ordered to the vulva, hip vapour-baths to be used night and morning, and sinapisms to be applied to the feet and thighs: no benefit was however obtained. Dr. R. had now recourse to the secale cornutum. Two drachms were infused in a litre (about two pints) of boiling water, for the space of an hour; a small coffee cup-ful was ordered to be taken every half hour: the effect seems to have been very speedy; for we are told "que la mere de la malade, trouva comme moi, une notable difference dans la vitesse du pouls"! within the first hour. (English physicians are not much in the habit of confirming their observations by the testimony of either the "pere" or the "mere.") Let it not be supposed, after this, (we transcribe the "ipsissima verba" of the doctor) that the action of the secale cornutum is gradual and tedious, or that it operates more on the womb than on the entire nervous system. The frequency of the dose was diminished in proportion as the pulse became slower and slower. A dose every two or three hours was after a short time deemed sufficient.

The evening paroxysms of pyrexia were arrested, and signs of general improvement were speedily visible. Fifteen days after the commencement of the remedy, the catamenia returned; and although their subsequent recurrence was not quite regular, the patient's health became most satisfactorily re-established.

*Case* 2. A robust healthy young woman had for five or six years been very irregular; before each period she suffered much from intense headache, congestive fullness of the neck, vomiting, and severe colic. Venesection and the other usual means resorted to in such cases brought only temporary relief. The secale cornutum was therefore ordered before an expected period: a drachm to be infused in a litre of water, and a small cupful to be taken every hour: on the second morning the menstrual discharge was induced, and continued for several days. For several successive periods this treatment was adopted, and always with entire success.

*Case* 3. A lady, 23 years of age, of a very nervous temperament, and hitherto "bien reglée," received a severe shock to her feelings by a melancholy accident to a friend at a time when she was unwell; the catamenia were immediately arrested; and from that period her general health became seriously indisposed. For several months there was no reappearance of the menses. Dr. R. prescribed the secale cornutum as in the preceding case: the medicine was taken in repeated doses for two days: most of the nervous symptoms were very speedily relieved, and at the end of a week the catamenia were induced. This patient had recourse to the ergot for several successive periods, and uniformly with the same satisfactory results.—*Journal Hebdomadaire.*

